# A relay velocity model infers cell-dependent RNA velocity

**DOI:** 10.1038/s41587-023-01728-5

**Published:** 2023-04-03

**Authors:** Shengyu Li, Pengzhi Zhang, Weiqing Chen, Lingqun Ye, Kristopher W. Brannan, Nhat-Tu Le, Jun-ichi Abe, John P. Cooke, Guangyu Wang

**Affiliations:** 1 Center for Bioinformatics and Computational Biology, Houston Methodist Research Institute, Houston, TX USA; 2 Center for Cardiovascular Regeneration, Houston Methodist Research Institute, Houston, TX USA; 3 Center for RNA Therapeutics, Houston Methodist Research Institute, Houston, TX USA; 4 Department of Cardiothoracic Surgery, Weill Cornell Medicine, Cornell University, New York, NY USA; 5 Department of Physiology, Biophysics & Systems Biology, Weill Cornell Graduate School of Medical Science, Weill Cornell Medicine, Cornell University, Ithaca, NY USA; 6 Department of Cardiology, The University of Texas MD Anderson Cancer Center, Houston, TX USA

**Keywords:** Computational models, Software, Data mining, Development, Sequencing

## Abstract

RNA velocity provides an approach for inferring cellular state transitions from single-cell RNA sequencing (scRNA-seq) data. Conventional RNA velocity models infer universal kinetics from all cells in an scRNA-seq experiment, resulting in unpredictable performance in experiments with multi-stage and/or multi-lineage transition of cell states where the assumption of the same kinetic rates for all cells no longer holds. Here we present cellDancer, a scalable deep neural network that locally infers velocity for each cell from its neighbors and then relays a series of local velocities to provide single-cell resolution inference of velocity kinetics. In the simulation benchmark, cellDancer shows robust performance in multiple kinetic regimes, high dropout ratio datasets and sparse datasets. We show that cellDancer overcomes the limitations of existing RNA velocity models in modeling erythroid maturation and hippocampus development. Moreover, cellDancer provides cell-specific predictions of transcription, splicing and degradation rates, which we identify as potential indicators of cell fate in the mouse pancreas.

## Abstract (web summary)

cellDancer enables RNA velocity estimation with cell-specific kinetics.

## Main

A cell may transition to a new fate during or after development in response to transcriptional factors and epigenetic modifiers that are modulated by intracellular or external signaling^1–5^. The advent of single-cell RNA sequencing (scRNA-seq) generated insights into cell subpopulations, detecting biological factors that influence cellular state shifts and deciphering cellular response to environmental and immune stimuli in health and disease at single-cell resolution^6,7^. High-throughput scRNA-seq data provide an unbiased and high-resolution transcriptomic landscape of cellular states^8^. However, scRNA-seq captures only snapshots of a set of cells and does not explicitly demonstrate dynamical transitions between cellular states. Thus, trajectory inference algorithms were developed by constructing a potential branching trajectory based on the similarity in the transcriptomic profiles^9–11^. A major challenge of trajectory inference is to determine the direction of the trajectories or the root and terminal cellular states. One way of inferring such directed dynamics of cellular states is to incorporate ‘RNA velocity’^12^. RNA velocity correlates the abundance of the nascent, unspliced mRNAs with that of the mature, spliced mRNAs using a simple first-order kinetics model. The progression of the current cellular state shifting toward a future state is extrapolated using the RNA velocities across genes. RNA velocity has brought biological insights to cell differentiation and disease progression^13–16^.

RNA velocity was proposed to model the dynamic process of transcription, splicing and degradation of mRNA in a single cell. This model was initially applied to circadian-associated genes to extrapolate the progression of the circadian cycle (24 hours) on the bulk RNA-seq data of the mouse liver^12^. Later, it was applied to infer the cell fates from scRNA-seq data, assuming that all cells in an scRNA-seq experiment share similar kinetics^12,17^. However, cellular state transitions often involve multiple stages and/or lineages, each of which may have dissimilar kinetics. The existing velocity models assume uniform kinetics of all cells in an scRNA-seq experiment, which may result in poor predictive performance when cell subpopulations have dissimilar RNA velocity kinetics. For example, a number of genes (for example, *Hba-x*) exhibit a boost in their transcription rates during mouse erythrocyte maturation, which have opposite predictions by scVelo^18^. It was also reported that there are five major branching lineages during the development of the mouse hippocampus^13^. The expression of some genes (for example, *Ntrk2*), termed branching genes, increase rapidly in several lineages and slowly in the other lineage. RNA velocities inferred by the existing models^12,17^ were inverted, in whole or in part, for the branching genes^18^. Thus, the estimation of RNA velocity kinetics is sensitive to heterogeneity in terms of biological conditions and cell populations.

**Fig. 1:**
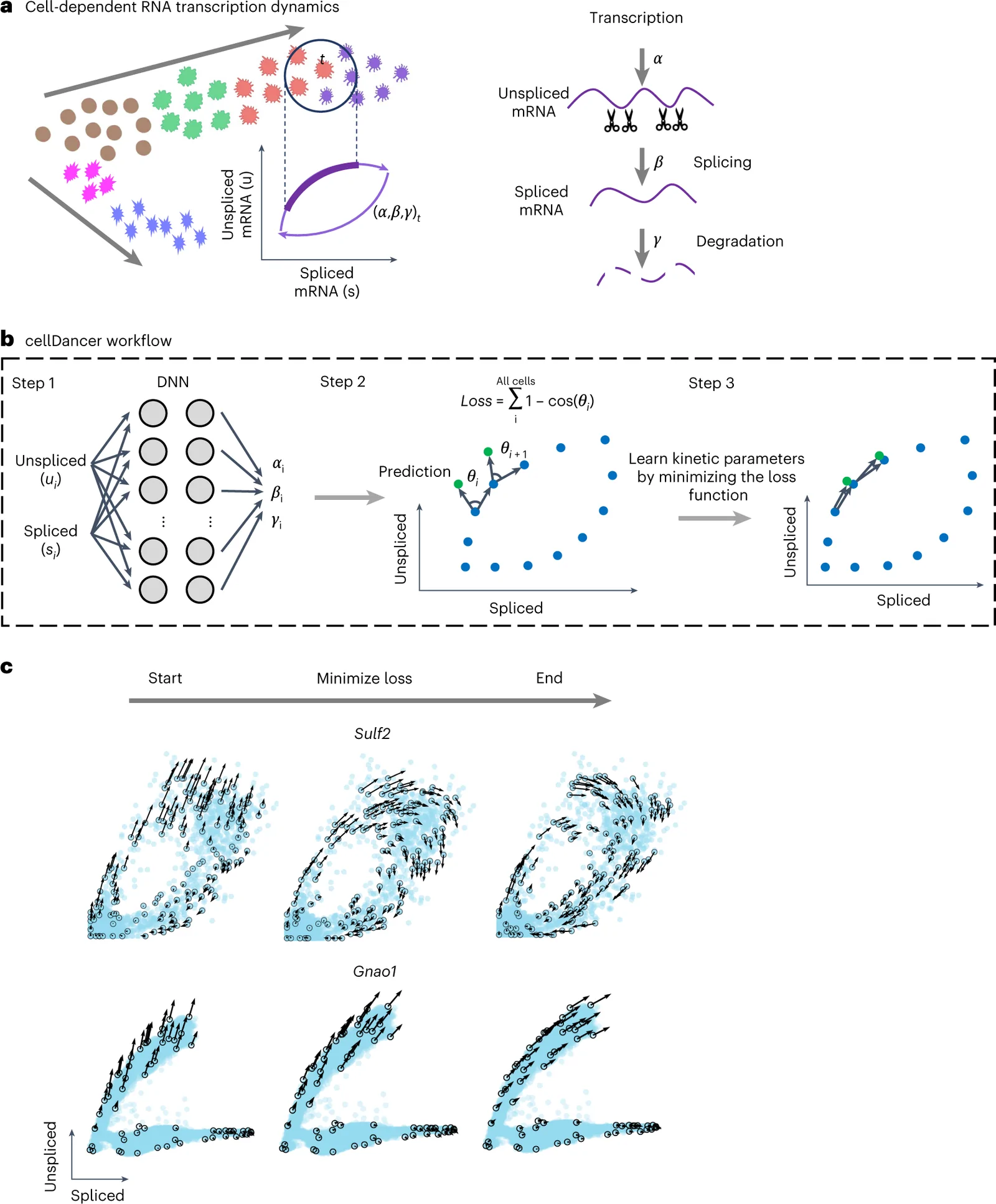
Predicting RNA velocity in localized cell populations via DNNs. **a**, Transcription dynamics of the premature (unspliced) and mature (spliced) mRNAs are governed by the transcription (*α*), splicing (*β*) and degradation (*γ*) rates. Multi-kinetics genes involve multiple-lineage and/or multi-stage transitions of the cellular states; hence, cell-dependent rates (*α*, *β*, *γ*)_*t*_ are required to accurately capture the transcription dynamics of those genes. In the illustration, the (*α*, *β*, *γ*)_*t*_ for cell *t* are computed by locating the future state cell in the neighboring cells of *t* (‘local environment’), assuming that the cells in the local environment share the same (*α*, *β*, *γ*). **b**, cellDancer uses a DNN to predict cell-specific *α*, *β* and *γ* for each gene. The DNN consists of an input layer with the spliced and unspliced mRNA abundances (*u*_*i*_, *s*_*i*_) *i* = 1,2, …, *n*_cells_, two fully connected hidden layers each with 100 nodes and an output layer yielding cell-specific *α*, *β* and *γ*. The loss function is defined as the sum of every cell’s cosine similarity of predicted and observed velocity vectors. The DNN is iteratively optimized by minimizing the loss function. **c**, The progress of minimizing the loss function. RNA velocities for the examples of the mono-kinetic gene *Sulf2* in pancreatic endocrinogenesis, and the multi-lineage gene *Gnao1* in mouse hippocampus maturation is projected onto the phase portraits during the training process of their DNNs.

Here we propose a ‘relay velocity model’ that uses the relay of a series of local velocities to provide single-cell resolution inference of velocity kinetics (Fig. 1a). Compared to other kinetic models, in the relay velocity model the cell-specific velocity of each cell is informed by its neighbor cells and then relays cell-specific velocities. To implement the relay velocity model, we developed cellDancer, which is a model-based deep neural network (DNN) framework. The cellDancer algorithm separately trains a DNN for each gene. For a gene, cellDancer assesses the spliced and unspliced mRNA velocities of each cell in a DNN to calculate the cell-specific transcription, splicing and degradation rates (*α*, *β* and *γ*) and to predict the future spliced and unspliced mRNA by the outputted *α*, *β* and *γ* using an RNA velocity model. The key step of cellDancer DNN is to define a loss function to train the DNN based on the similarity between the predicted future spliced and unspliced mRNA of each cell and the observation of its neighbor cells. After optimizing the global similarity between prediction and observation, cellDancer infers *α*, *β* and *γ* at a single-cell resolution rather than bulk rates used in existing methods^12,17^.

We demonstrate that cellDancer extends the velocity estimation with cell-specific kinetics on heterogeneous cell populations, including those involved in erythroid maturation during gastrulation and those of the hippocampal dentate gyrus during neurogenesis. The cellDancer algorithm outperforms steady and early switching models on multi-stage and multi-lineage cell subpopulations. We show that cell-specific *α*, *β* and *γ* could be indicators of fate for cell identity in the mouse pancreas. cellDancer is available as a highly modularized, parallelized and scalable implementation.

## Results

### Learning cell-specific RNA kinetics by a relay velocity model

The cellDancer algorithm is a deep learning framework to generalize the estimation of RNA velocity in both homogeneous and heterogeneous cell populations from scRNA-seq data by estimating cell-dependent transcription (*α*), splicing (*β*) and degradation (*γ*) rates. Cell-specific *α*, *β* and *γ* were predicted by an RNA velocity model that incorporated the neighbor cells (see details regarding the selection of the neighbor cells in the Methods). Specifically, we resolved the RNA velocity kinetics by estimating the reaction rates from the weights and biases of the nodes in a DNN, which is a generalized framework of velocity estimation (see a demonstration in Supplementary Note 1). To train the cellDancer DNN, we first discretized the original reaction kinetics as follows:

$$\begin{array}{rcl}\displaystyle\frac{{u\left( {t + \Delta t} \right)\, -\, u\left( t \right)}}{\Delta t} & = & \alpha \left( t \right) - \beta \left( t \right)u\left( t \right),\\ \displaystyle\frac{{s\left( {t + \Delta t} \right)\, - \,s\left( t \right)}}{{\Delta t}} & = & \beta \left( t \right)u\left( t \right) - \gamma \left( t \right)s\left( t \right),\end{array}$$

where time *t* is discretized and Δ*t* is a small time slot. In our model, *α*, *β* and *γ* are cell specific. For an individual gene in cell *i*, cellDancer used a DNN to predict cell-specific rates *α*(*t*_*i*_), *β*(*t*_*i*_) and *γ*(*t*_*i*_) from the spliced and unspliced mRNA abundances *u*(*t*_*i*_) and *s*(*t*_*i*_) of genes at time *t* and neighboring cells of *i* (Fig. 1b). Second, we extrapolated *s*(*t*_i_ + Δ*t*) and *u*(*t*_*i*_ + Δ*t*) of cell *i* at time *t* + Δ*t* to infer a velocity vector that points from the current state to the future in the gene phase portrait. We defined a loss function by summing every cell’s maximum cosine similarity for the predicted and observed velocity vectors (Methods). Finally, optimized rates of each cell were obtained by minimizing the loss function (Fig. 1b).

**Extended Data Fig. 1:**
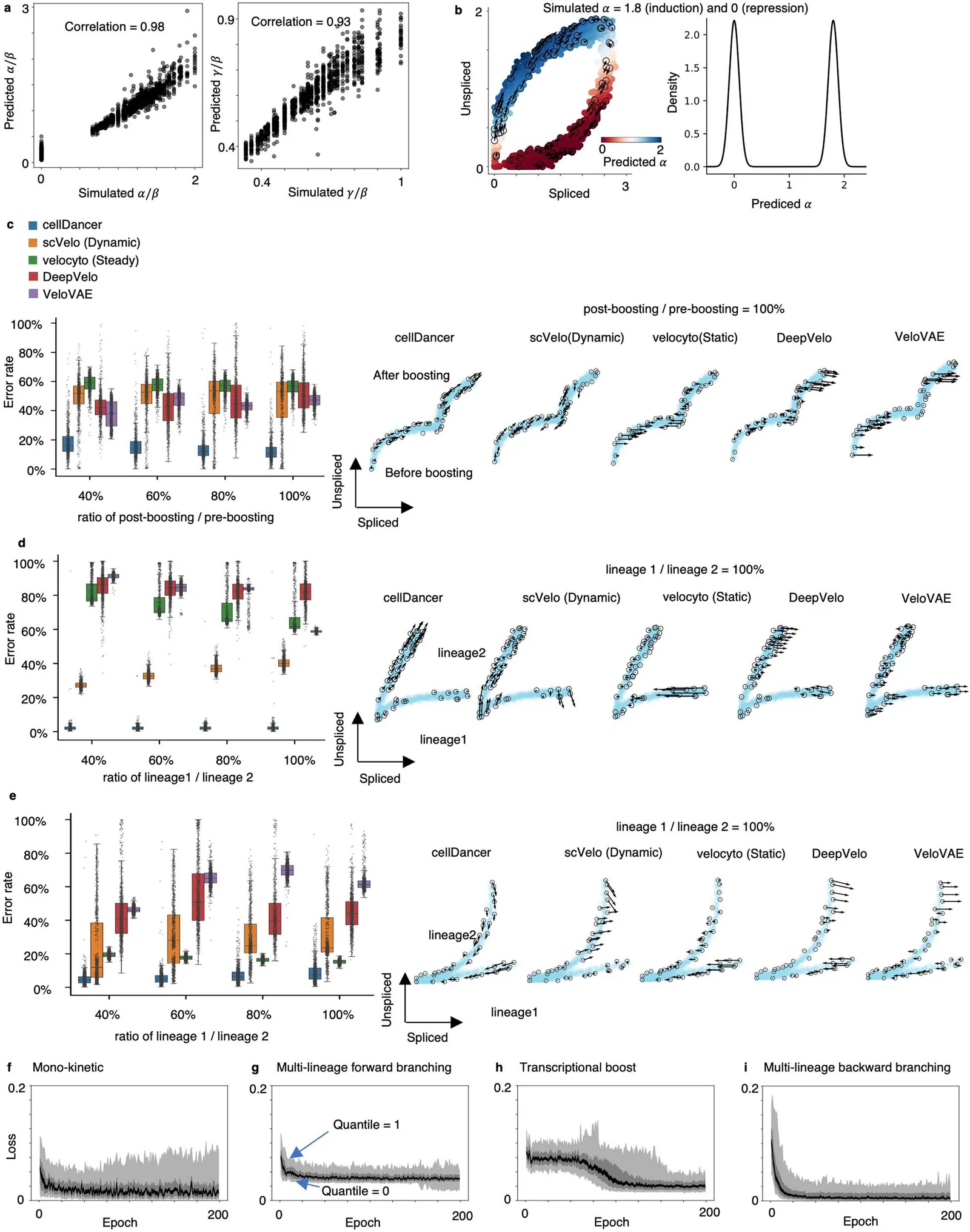
Resolving RNA velocity of simulated multiple rate kinetics genes. **(a)** High correlation between the simulated (background truth) and the predicted 𝛼/𝛽 (left) and 𝛾/𝛽 (right). The Pearson correlation coefficients (R^2^) between the prediction and the simulation are computed. **(b)** RNA velocity predicted by cellDancer is projected onto the phase portraits of a simulated gene with 𝛼 equals 1.8 (induction) and 0 (repression) (left) and the density plot of the predicted 𝛼 (right). (c-e) We measured the accuracy by computing the error rate as the percentage of cells whose predicted RNA velocity is poorly correlated with the ground truth velocity (cosine similarity < 0.7). The box plots of the error rates show that cellDancer outperforms scVelo, velocyto, DeepVelo, and VeloVAE in the estimation of RNA velocities for the simulated transcriptional boost **(c)**, multi-lineage forward branching **(d)**, and multi-lineage backward branching **(e)** genes. Middle line in box plot, median; box boundary, interquartile range; whiskers, 10–90 percentile; minimum and maximum, not indicated in the box plot; gray dots, individual datapoints. The error rate is defined as the percentage of falsely predicted directions. Different sampling ratios were investigated at 40%, 60%, 80%, and 100% (n = 1,000 genes), representing the ratio of the number of post-boosting cells to the number of pre-boosting cells in **(c)** and the ratio of the number of cells in lineage 1 to the number of cells in lineage 2 (d-e). Example phase portraits for sampling ratio (1:1) are provided in each case. (f-i) The loss scores are plotted against epochs of training on the simulated mono-kinetic (top left), multi-lineage forward branching (top right), transcriptional boost (bottom left), and multi-lineage backward branching (bottom right) genes at quantiles 0, 0.1, 0.4, 0.6, 0.9, and 1. In all cases, the loss scores converge in about 25 epochs, except for the transcriptional boost genes, for which the convergence emerges in about 100 epochs.

We initially evaluated the training progress of cellDancer on several well-studied genes in pancreatic endocrinogenesis and mouse hippocampus development^17^. We observed that cellDancer captured the transcriptional dynamics of these genes (Fig. 1c and Supplementary Fig. 1). Then, we scaled up the performance evaluation of cellDancer on 1,000 simulated mono-kinetic genes with the shared *β*, *γ* and two-step *α* values. The predicted parameters are highly correlated with the ground truth (*R*^2^ = 0.98 for *α*/*β* and 0.93 for *γ*/*β*; Extended Data Fig. 1a). Remarkably, cellDancer can identify two clusters of *α* values representing active (positive) and repressive expression phases (centered ~0) on a benchmark dataset, without a prior constraint of a two-step transcription rate (Extended Data Fig. 1b).

### Inferring RNA velocity in multi-rate kinetics

As cellDancer provides the single-cell resolution of *α*, *β* and *γ*, we next examined whether cellDancer could resolve the multi-rate kinetic regimes. We simulated three multiple kinetic regimes, including transcriptional boost, multi-lineage forward and multi-lineage backward genes (Extended Data Fig. 1c–e, right panels, and Methods). Transcriptional boost refers to a boost in the expression induced by a change in the transcription rate; multi-lineage forward and multi-lineage backward refer to induction and repression in separate lineages, respectively. We generated 2,000 cells and 1,000 genes for each regime. We compared cellDancer with scVelo (dynamic) and velocyto (static) algorithms and two deep learning algorithms, DeepVelo^19^ and VeloVAE^20^. The error rates in cellDancer were significantly lower than those in scVelo, velocyto, DeepVelo and VeloVAE in all three simulated regimes (Extended Data Fig. 1c–e; *P* < 0.001, one-sided Wilcoxon test). Specifically, cellDancer exhibited the lowest error rate for simulated transcriptional boost, multi-forward branching and multi-backward branching kinetics with 13%, 3% and 9% compared to velocyto, scVelo, DeepVelo and VeloVAE, respectively (Supplementary Table 1). To test the effect of imbalanced cell numbers in different lineages or stages, we downsampled the cells at the stage after transcriptional boosting (Extended Data Fig. 1c) and the cells in lineage 1 (Extended Data Fig. 1d,e). Results showed that cellDancer is not affected by the bias of cell distribution. Next, we estimated the required number of epochs to optimize cellDancer DNN. cellDancer converged at 25 epochs for mono-kinetic, multi-forward and multi-backward branching genes and 100 epochs for transcriptional boost genes (Extended Data Fig. 1f–i).

### Delineating transcriptional boost on single-cell resolution

**Fig. 2:**
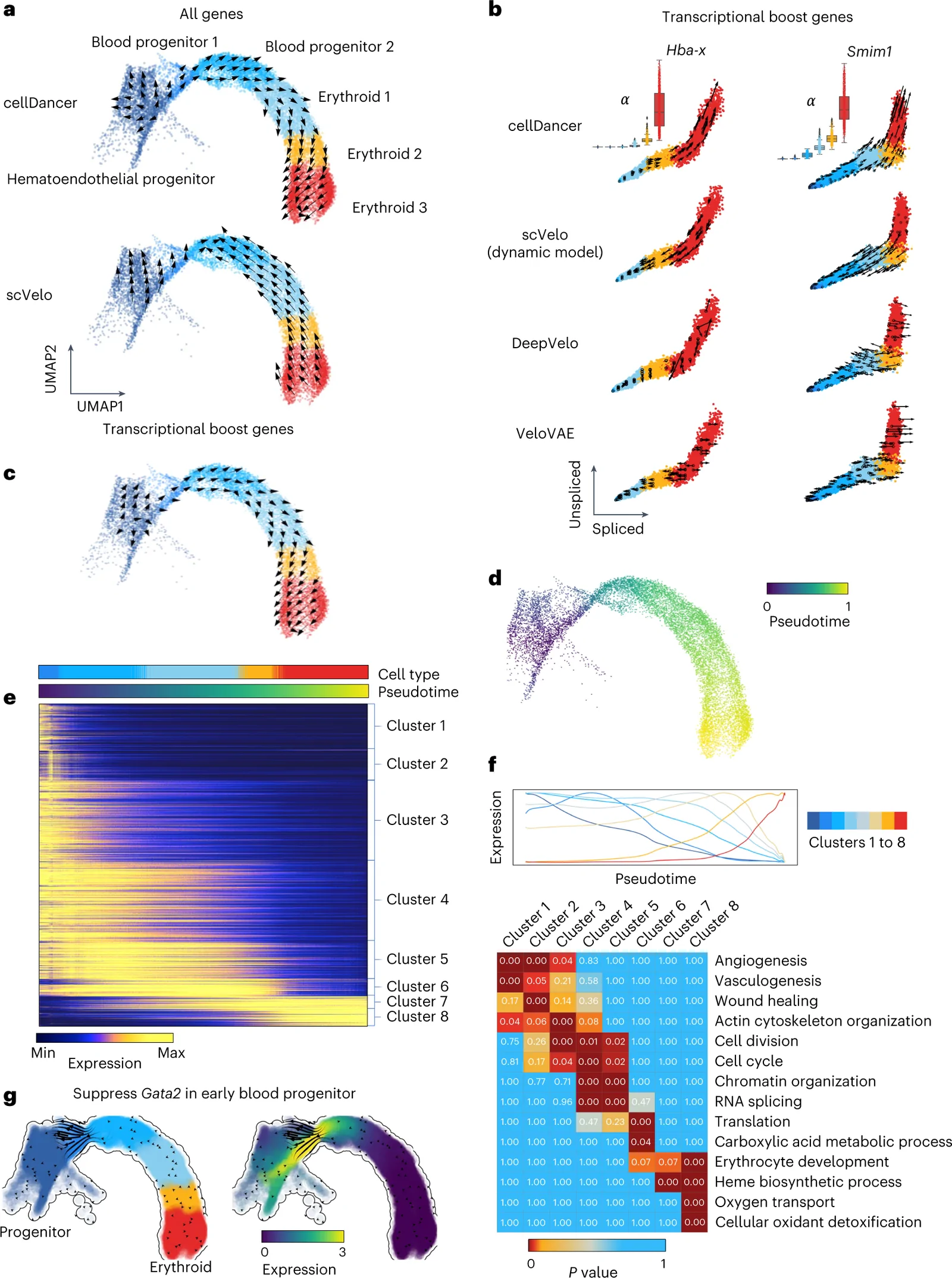
Delineating gastrulation erythroid maturation and resolving transcriptional boost. **a**, The velocities derived from cellDancer (top) are consistent with the erythroid differentiation but opposite in scVelo dynamic model (bottom) by using all genes. **b**, The velocities derived from cellDancer, scVelo dynamic model, DeepVelo and VeloVAE for the transcriptional boost genes (*Hba-x* and *Smim1*) are illustrated on the phase portraits. The cells are colored according to the cell types. The box plots of *α* for each cell type predicted by cellDancer are included to show the boost in the *α* rates in the course of erythroid maturation, especially in erythroid 3. **c**, The velocities derived from cellDancer for gastrulation erythroid maturation using transcriptional boost genes are projected on the UMAP of the original work, demonstrating that cellDancer can infer the correct cell differentiation direction by using only the transcriptional boost genes. **d**, Gene-shared pseudotime on UMAP is consistent with the progression of gastrulation erythroid maturation. **e**, Genes that show high similarity in transcriptional changes along time are classified into eight clusters according to their transcriptional changes. The heat map describes the expression of the genes along time (rows: genes; columns: cells ordered according to the pseudotime). Genes were selected by Pearson correlation coefficient (*R*^2^) > 0.8. **f**, Average expression of each cluster along the pseudotime (top) and the enriched pathways for each cluster of genes (bottom) (Benjamini–Hochberg procedure, one-sided, *P* < 0.05). *P* value indicates the significance of enrichment of a pathway in Fisher’s exact test. **g**, In silico perturbation analysis by dynamo shows a critical role of *Gata2* in hematopoiesis.

**Extended Data Fig. 2:**
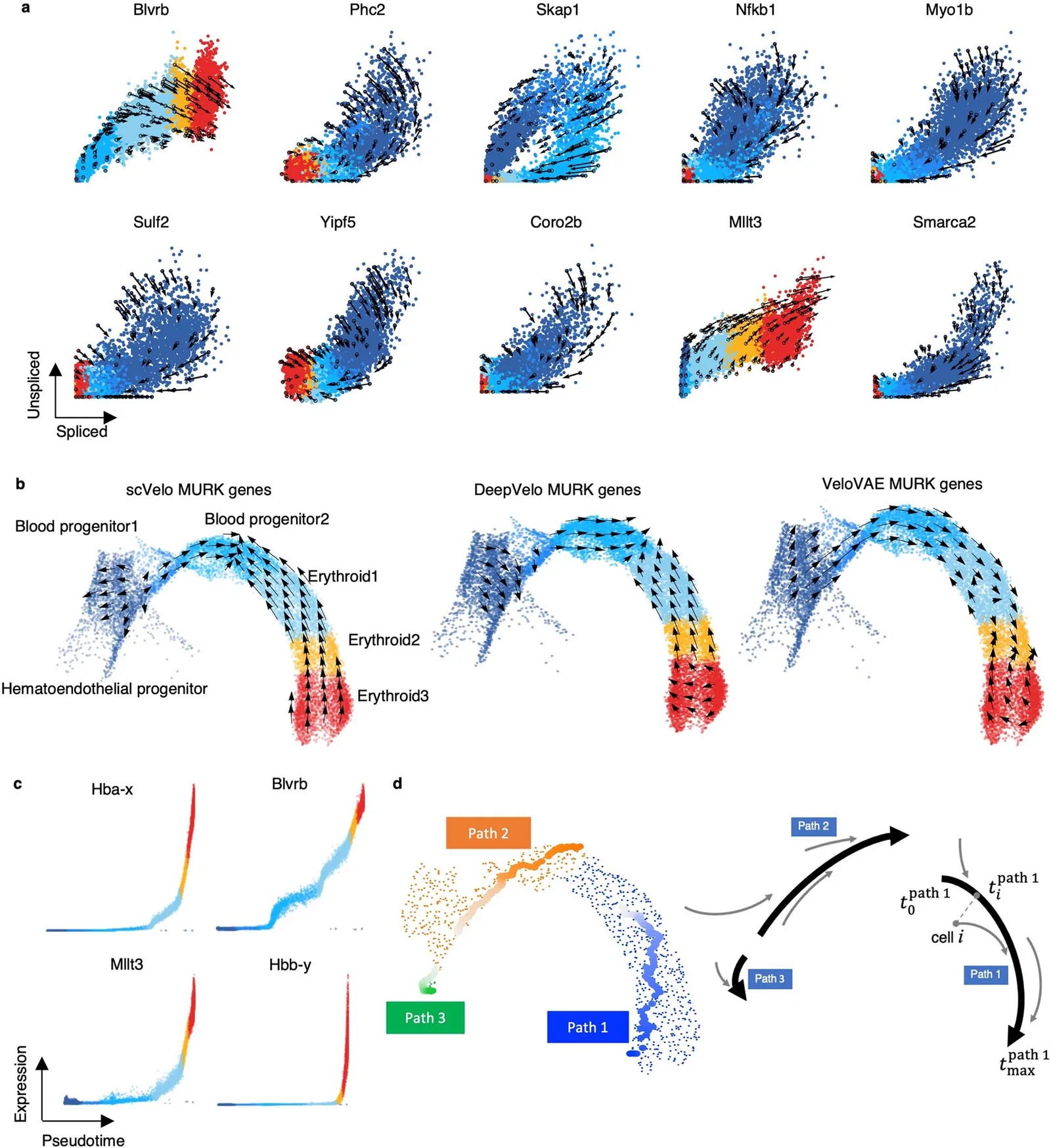
RNA velocity estimation for the multiple rate kinetics (MURK) genes in the gastrulation erythroid maturation dataset. **(a)** RNA velocities predicted by cellDancer are projected onto the spliced-unspliced phase portraits for a set of selected genes. **(b)** The velocities derived from scVelo dynamic model, DeepVelo, and VeloVAE for gastrulation erythroid maturation cells using MURK genes are visualized on the pre-defined UMAP embedding. Inverted flows from the erythroid 3 to the blood progenitors 2 cell type are observed for the scVelo and DeepVelo predictions; inverted flows from the erythroid 3 to the erythroid 1 cell type are observed for the VeloVAE prediction. **(c)** Expression pseudotime profiles for four MURK genes *Hba-x*, *Blvrb*, *Mllt3*, and *Hbb-y* show the expression patterns of transcriptional boost in gastrulation erythroid maturation. **(d)** Long trajectories used for pseudotime estimation in gastrulation erythroid maturation are visualized on the UMAP embedding. The long trajectories are local maxima of traverse length and are colored from light to dark according to their unadjusted pseudotime. The schematic diagram demonstrates how the unadjusted pseudotime of cells is determined according to the time in the long trajectories. The black bold lines stand for the long trajectories and the pseudotime for the originating cells of the short trajectories (gray lines) is obtained as the time of the closest cell in the corresponding long trajectory.

We compared cellDancer to the dynamical model of scVelo on the scRNA-seq experiment of mouse gastrulation erythropoiesis^2^ (Extended Data Fig. 2a and Fig. 2a), in which transcriptional boost genes were reported^13^. The vector flow in a uniform manifold approximation and projection (UMAP) embedding of the transcriptome clearly suggests that cellDancer recaptures the progression of erythroid differentiation (Fig. 2a, top), whereas scVelo’s prediction was reversed^18^ (Fig. 2a, bottom).

Barile et al.^18^ identified 89 multiple rate kinetics (MURK) genes, such as *Smim1* and *Hba-x*, of which transcription rates boost in the middle of erythroid differentiation, and showed that the prediction of scVelo was severely affected by the boost of transcription, resulting in incorrect predicted directions. cellDancer predicted the correct changes of well-known MURK genes, such as *Smim1* and *Hba-x*, on the phase portraits (Fig. 2b), whereas scVelo, DeepVelo and VeloVAE had incorrect predictions. Moreover, cellDancer revealed the transcriptional boost by the cell-specific *α* (Fig. 2b). We next tested the overall prediction of cellDancer on transcriptional boost genes. We applied cellDancer and scVelo to the 89 MURK genes and projected the velocity inference to the transcriptome UMAP. cellDancer recaptured the correct directional flow of differentiation using only MURK genes (Fig. 2c), whereas scVelo, DeepVelo and VeloVAE predicted an opposed direction in multiple cell types (Extended Data Fig. 2b).

Next, we demonstrated cellDancer’s capabilities of deciphering transcriptional changes along the differentiation pseudotime. We first inferred major trajectories during cell differentiation from the transition matrix based on the correlation of velocities among neighbor cells (Methods). Then, we estimated a universal pseudotime from trajectories to capture the cell’s position along with the erythroid maturation. The pseudotime of cellDancer accurately illustrated the transcriptional changes of genes (Extended Data Fig. 2c) and the terminal of erythroid maturation (Fig. 2d). To delineate the dynamics of transcriptional activity, we grouped genes into eight clusters based on the similarity in the transcriptional changes along pseudotime (Fig. 2e). The expression of genes in the first three clusters was high at the early stage in the hematoendothelial progenitor cells and diminished during differentiation. Gene expression in clusters 4–6 decreased slower than the gene expression in the first three clusters and decreased close to zero in the erythroid 3 subpopulation. Gene expression in clusters 7 and 8 increased during erythroid maturation. We next investigated the biological function of each gene cluster during erythroid cell differentiation. Gene Ontology (GO) analysis through DAVID^21^ showed that these genes are highly enriched in the angiogenesis and wound healing pathways. Genes in clusters 4–6 were enriched in basic cellular functions, including cell cycle, cell division, chromatin organization, RNA splicing and translation pathways. It is not surprising that these genes are enriched in erythrocyte development, heme biosynthetic process, oxygen transport and cellular oxidant detoxification pathways (Fig. 2f). Finally, we applied dynamo^22^ to in silico suppress the expression of *Gata2*, a critical regulator in hematopoiesis, in blood progenitor 1. We observed the diversions of hematopoietic fate after the perturbation (Fig. 2g), which is consistent with the experimental study^23^.

### Inferring RNA velocities on each branch for branching genes

**Fig. 3:**
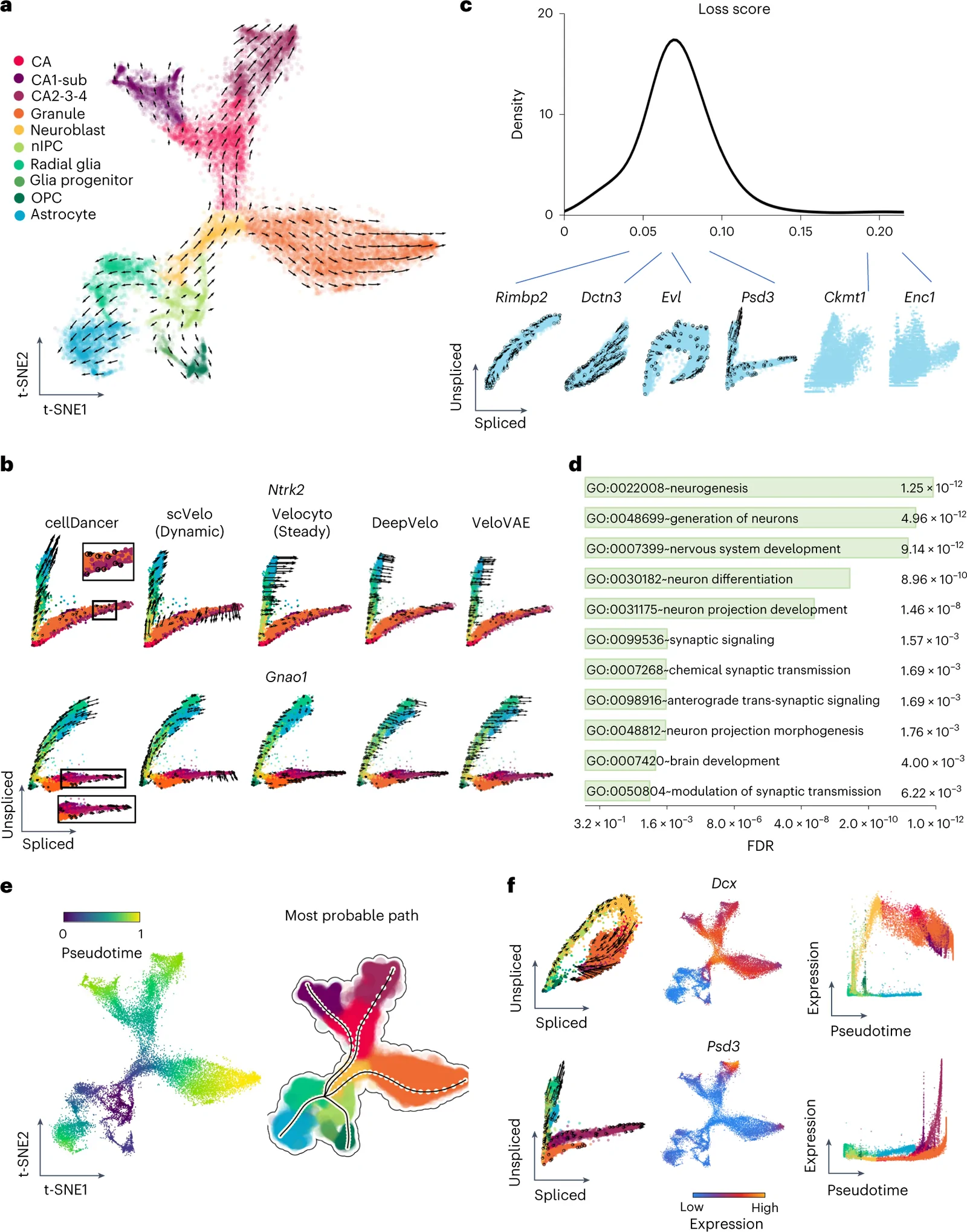
Identifying the branching lineage in the hippocampus development. **a**, The velocities derived from cellDancer for the mouse hippocampus development dataset are visualized on the pre-defined t-SNE embedding. Directions of the projected cell velocities on t-SNE are in good agreement with the reported directions. **b**, The phase portraits of two branching genes (*Ntrk2* and *Gnao1*) predicted by cellDancer, scVelo dynamic mode, velocyto, DeepVelo and VeloVAE demonstrate the advantage of cellDancer in predicting the velocities of the branching genes. The RNA velocities of *Ntrk2* and *Gnao1* predicted by cellDancer are consistent with the expectation of hippocampus developmental progress, whereas the directions predicted by others are inconsistent in part. The cells are colored according to the cell types. **c**, Distribution of the minimized loss for all the genes. Those genes with low loss scores show mono-kinetic or divergent dynamics, whereas genes with high loss scores show pattern-less phase portraits. **d**, The GO pathway enrichment analysis using adjusted *P* values of Fisher’s exact test (Benjamini–Hochberg procedure, one-sided, *P* < 0.05) of DAVID for the 500 genes with the lowest training loss score shows that these genes are highly involved in pathways associated with nervous and brain development. **e**, Gene-shared pseudotime is projected on t-SNE by cellDancer, and the most probable paths are inferred by dynamo, showing the order of cell differentiation during hippocampus development. **f**, The phase portraits (left, cells colored according to **a**), the expression on t-SNE embedding (middle) and the expression pseudotime profiles (right) for the genes *Dcx* and *Psd3*. *Dcx* (top) and *Psd3* (bottom) have distinct dynamic behaviors. *Dcx* is a mono-kinetic gene (left), and its expression gradually increases in neuroblasts (right). *Psd3* is a branching gene (left), and its expression increases in each branching lineage at different speeds (right). FDR, false discovery rate; nIPC, neural intermediate progenitor cell.

We evaluated cellDancer using data from the branching lineages in mouse hippocampus development. There are five major branching lineages in the mouse hippocampus, corresponding to dentate gyrus granule neurons, pyramidal neurons in subiculum and CA1, pyramidal neurons in CA2/3/4, oligodendrocyte precursors (OPCs) and astrocytes^12^. The cell velocity graph shows that cellDancer accurately inferred five major branching lineages in hippocampus development (Fig. 3a), confirming the reliable performance of cellDancer on multi-lineage populations.

**Extended Data Fig. 3:**
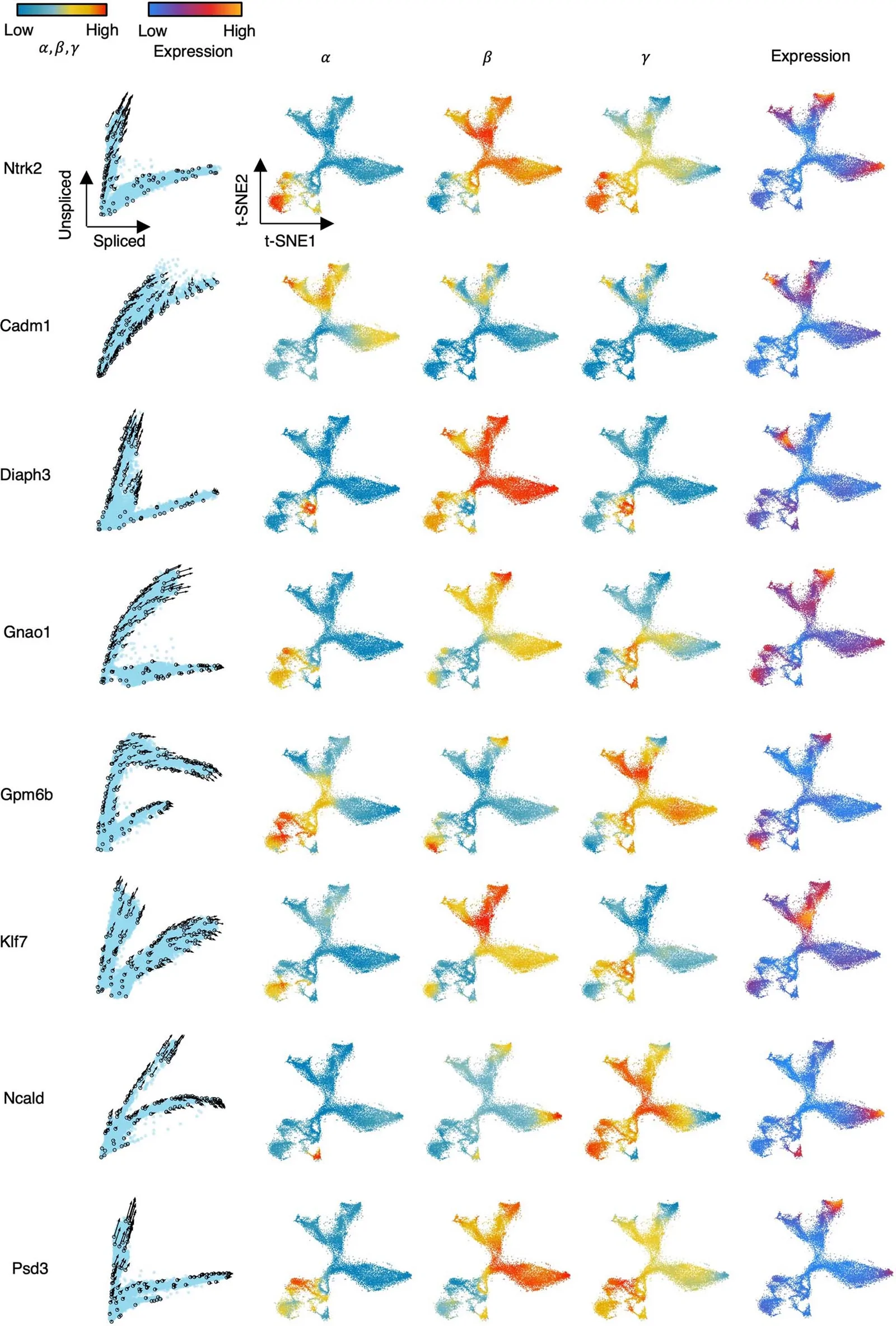
Cell-specific kinetic rate parameters improve RNA velocity inference in hippocampus development. Panel (1) Velocities of selected genes inferred by cellDancer are projected onto the phase portraits; Panels (2–4) Cells are colored according to the cell-specific *α*,*β*, and *γ* rates for the referenced gene in the t-SNE embedding for the hippocampus development; Panel (5) Cells are colored according to the gene expression.

We further studied the velocity inference of individual branching genes. As branching genes have different reaction rates among lineages, they have lineage-specific regulation of transcription, splicing and degradation and often play an important role in hippocampus development. For example, branching genes are vital to neurogenesis (*Diaph3*, *Klf7* and *Ncald*; Extended Data Fig. 3)^24–26^ and are involved in the differentiation of the neural system (*Cadm1* and *Gpm6b*)^27,28^. Branching genes are also related to neurological or neuropsychiatric disorders. For instance, mutations of *Gnao1* may contribute to epilepsy, developmental delay and movement disorders in the neural system^29^. Aberrant *Psd3* proteins are related to autism spectrum disorder and schizophrenia^30^. We applied cellDancer to the branching genes. Phase portraits show that cellDancer can accurately infer the velocities of branching genes on each lineage (Fig. 3b and Extended Data Fig. 3), whereas scVelo, velocyto, DeepVelo and VeloVAE predicted the correct velocities on a limited number of cells (Fig. 3b and Supplementary Fig. 2). Moreover, cell-specific *α*, *β* and *γ* were inferred on each branch. For instance, neurotrophic tyrosine kinase receptor type 2 (*Ntrk2*)^31^ has two major branches: the upper branch corresponds to astrocytes and OPCs, and the lower branch corresponds to dentate gyrus granule neurons and pyramidal neurons (Fig. 3b). Astrocytes and OPCs have high *α* and low *β*, resulting in high expression of unspliced *Ntrk2* on the upper branch. Dentate gyrus granule neurons and pyramidal neurons have high *β* and low *γ*, resulting in high expression of spliced *Ntrk2* on the lower branch (Extended Data Fig. 3).

cellDancer calculates a minimized loss function after optimizing a DNN for each gene. A small loss score indicates a good fit with the RNA velocity model. We ranked genes based on their loss function score. Top-ranking genes include both mono-kinetic and branching genes (Fig. 3c). Next, we performed GO pathway enrichment analysis through DAVID^21^ for the top 500 genes. The enriched pathways are associated with neurogenesis, nervous system development, neuron differentiation, synaptic signaling, chemical synaptic transmission and brain development (Fig. 3d).

We applied pseudotime analysis to infer the differentiation order of cells in hippocampus development. cellDancer automatically identified radial glia cells as a shared root state of hippocampus development (Fig. 3e), which is in good agreement with the previous study^32^. We also identified five terminal states without prior knowledge of the number of branches in the development process and applied dynamo to predict the most probable path of each terminal state (Fig. 3e). The pseudotime analysis of cellDancer suggests that astrocytes and OPCs are produced earlier than granule neurons and pyramidal neurons. Together, cellDancer has the capability to infer the global differentiation pseudotime of branching cell lineages.

**Extended Data Fig. 4:**
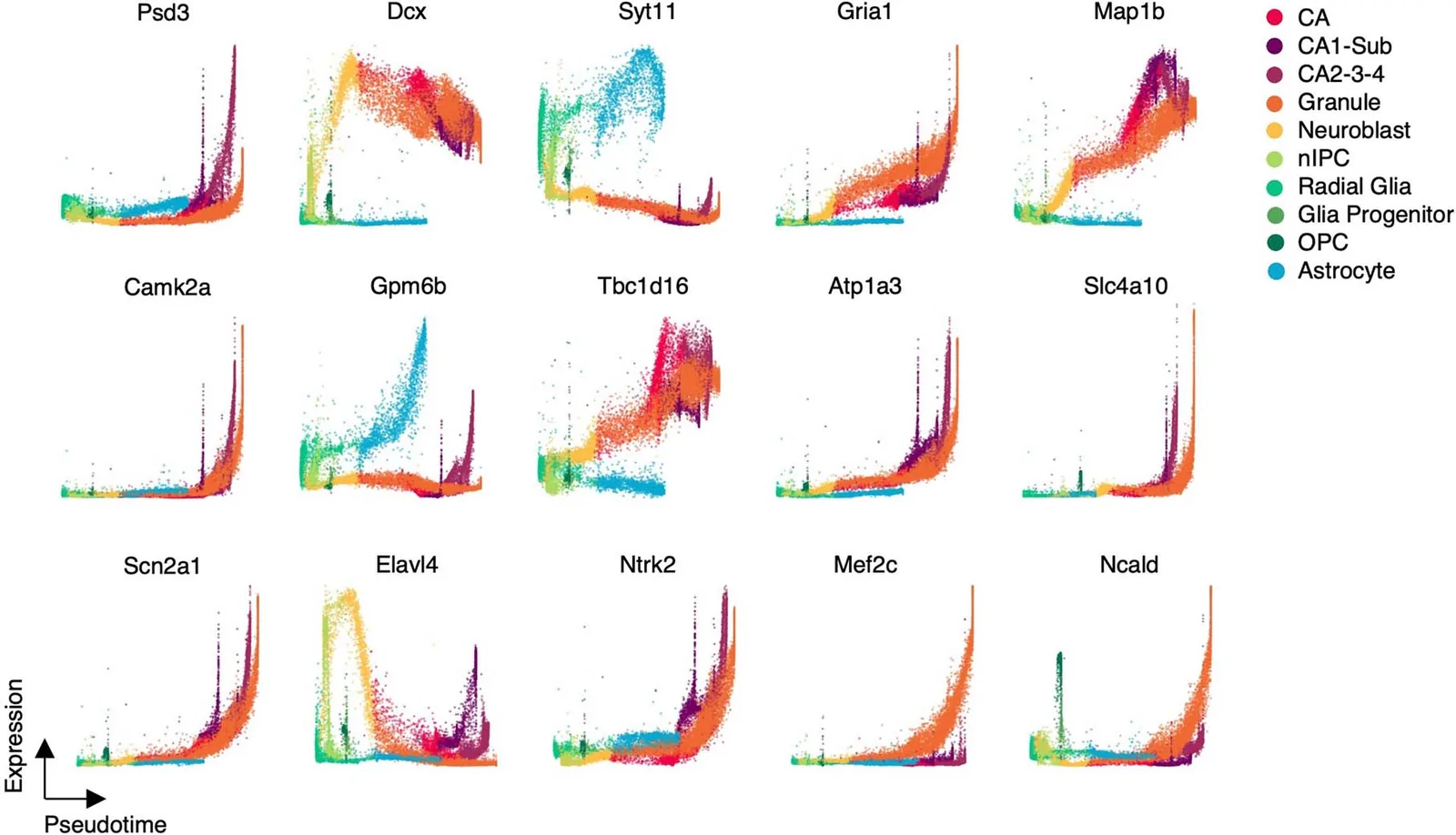
The expressions along pseudotime of genes in hippocampal neurogenesis data. The expression pseudotime profiles for a selected set of genes in hippocampal neurogenesis.

We investigated the temporal progression of transcription during hippocampus development. We observed multiple expression patterns of individual genes on different branches. For instance, *Dcx* transiently upregulates in neuroblasts with consistently low expression in astrocytes (Fig. 3f), which is supported by previous studies that *Dcx* transiently expresses in the early neurogenesis stage and is a widely used marker for neurogenesis^33,34^. By contrast, genes associated with neurogenesis, such as *Slc4a10* (ref. ^35^), *Ncald*^26^ and *Ntrk2* (ref. ^31^), show increasing expression in all branches at different rates (Extended Data Fig. 4).

### Vector fields analysis using cell-specific RNA velocity

**Fig. 4:**
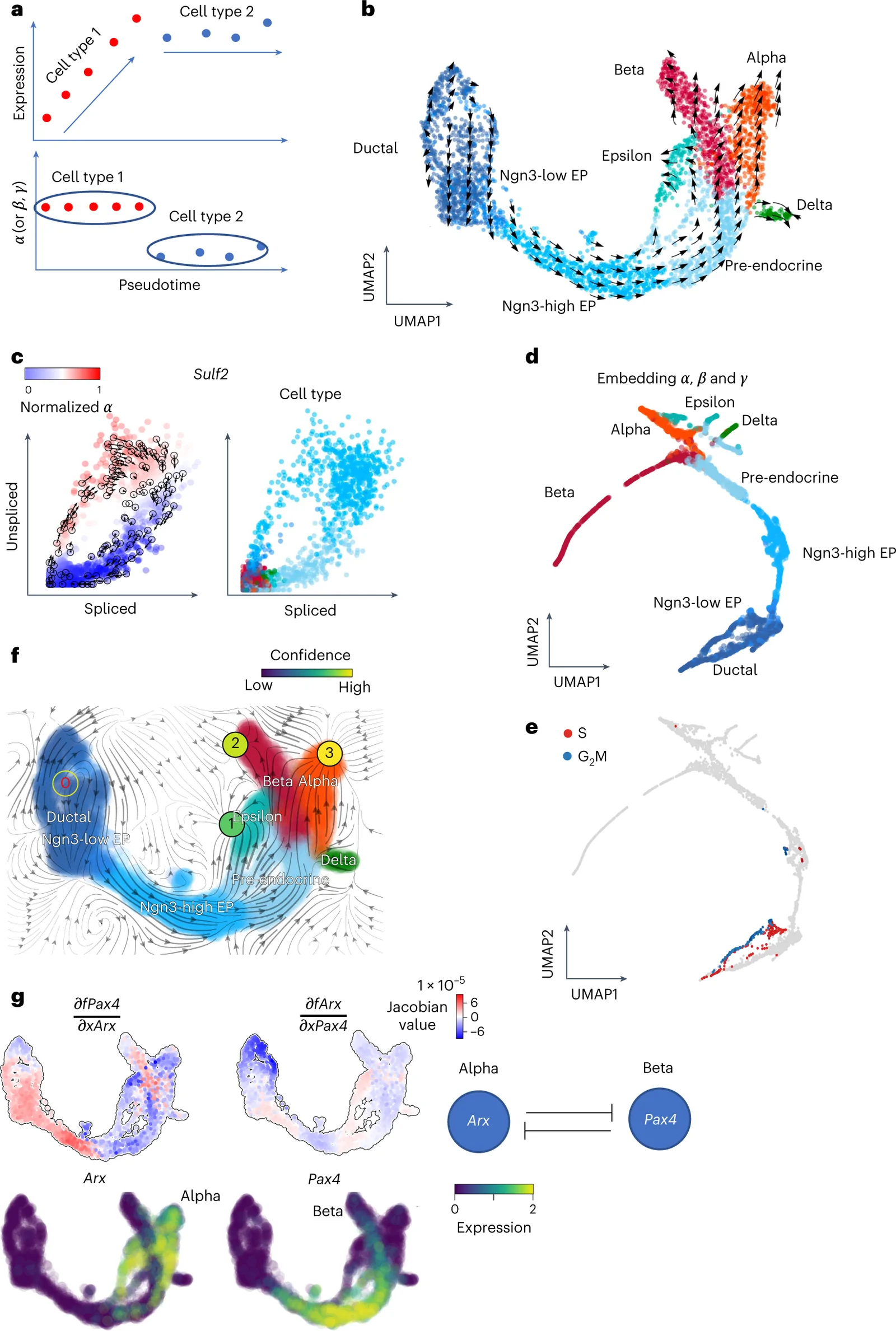
Deciphering cell identity with cell-specific reaction rates and analyzing gene regulation through vector fields. **a**, Schematic illustration shows that the *α*, *β* or *γ* rates of the genes may be a good indicator of the cell types rather than the expressions of the genes. **b**, The velocities derived from cellDancer for the pancreatic endocrinogenesis cells are visualized on the pre-defined UMAP embedding. **c**, Phase portraits of the gene *Sulf2*. The *α* rates of the *Sulf2* gene for each cell calculated by cellDancer clearly illustrate the gene’s induction and regression phases (left). *Sulf2* is in induction in the Ngn3-high embryonic progenitor (EP) cell type and in regression in the pre-endocrine cell type, whereas it is barely transcribed in other cell types (right). **d**,**e**, UMAP embedding using the cell-specific *α*, *β* and *γ* rates calculated by cellDancer indicates that our computed kinetics rates might be useful in assigning cell subpopulations (**d**) and cell identity (**e**). **f**, The velocity vector fields were learned by dynamo. The red digit 0 reflects the identified emitting fixed point. The black digits 1, 2 and 3 reflect the absorbing fixed points. **g**, Jacobian analysis and the gene expression of *Arx* and *Pax4* on the UMAP space. It shows that *Pax4* is downregulated by *Arx* in alpha-cells. *Arx* is downregulated by *Pax4* in beta-cells.

cellDancer extends the bulk reaction rates (*α*, *β* and *γ*) to single-cell resolution in an scRNA-seq experiment. As gene expression is regulated by transcription, splicing and degradation, the reaction rates tend to be more stable than expression in a cell type during cell differentiation (Fig. 4a). Thus, we asked if the cell-dependent reaction rates in cellDancer provide biological insights into cell identity. We applied cellDancer to infer cell-dependent *α*, *β* and *γ* in the endocrine development of the mouse pancreas profiled from embryonic day 15.5 (E15.5)^36^. Previous works reported four terminal cell types in endocrinogenesis, including glucagon-producing alpha-cells, insulin-producing beta-cells, somatostatin-producing delta-cells and ghrelin-producing epsilon-cells^37^. UMAP of transcriptome shows that alpha-, beta-, delta- and epsilon-cells are distributed closely (Fig. 4b). Reaction parameters are always more consistent than transcriptomes in a cell type. For instance, expression of *Sulf2* increases in Ngn3-low endocrine progenitors and decreases in pre-endocrine (Fig. 4c), whereas *α* is a similar positive value in Ngn3-low endocrine progenitors and ~0 in pre-endocrine. Next, we investigated the overall similarity of *α*, *β* and *γ* in each cell type. We applied UMAP to embed *α*, *β* and *γ* into two dimensions. Alpha-, beta-, delta- and epsilon-cells separate into distinct groups on UMAP of *α*, *β* and *γ* (Fig. 4d and Supplementary Fig. 3), suggesting that cell-specific *α*, *β* and *γ* are available as an indicator of cell identity. Notably, the cycling subpopulation of ductal cells and endocrine progenitors was separated from those without cycling (Fig. 4e).

Furthermore, we inputted the cell velocity to the established framework dynamo, which provides rich downstream analyses by learning differentiable velocity vector fields and inferring gene regulation networks. Noticeably, absorbing fixed points are identified in the alpha-, beta- and epsilon-cells, and an emitting fixed point is identified in the pancreas progenitor cells (Fig. 4f). To investigate the alpha-cell and beta-cell fate determination, we inspected the expression of *Arx* and *Pax4*, two well-known transcription factors that determine the endocrine cell fates (the alpha and beta lineages)^38^. Consistent with the previous study^38^, we observed exclusively high expression of *Arx* and *Pax4* in the alpha-cells and beta-cells, respectively (Fig. 4g). Then, we used dynamo to perform Jacobian analyses and detected mutual inhibition between *Arx* and *Pax4* in the alpha-cells and beta-cells. These analyses are in line with the experimental findings^39^ and provide mechanistic insight from gene regulation at single-cell resolution, showing that cellDancer can be seamlessly integrated with downstream analysis, such as dynamo vector field analysis.

### Revealing the turnover strategies of mRNA during cell cycle

**Extended Data Fig. 5:**
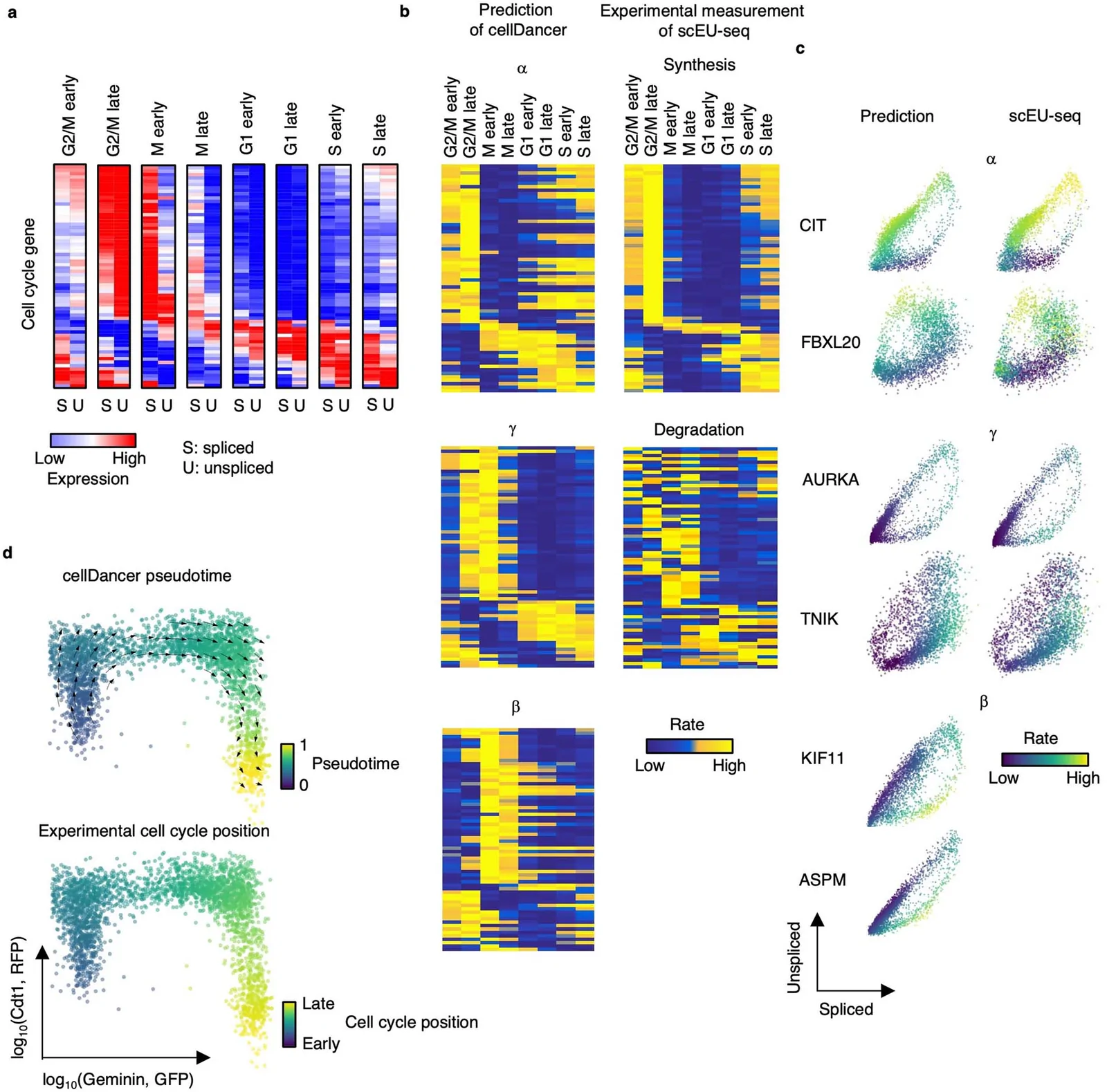
Revealing the turnover strategies of mRNA in the cell cycle process. **(a)** The spliced and unspliced reads of cell cycle genes in the cell cycle progression. The averaged spliced and unspliced reads were calculated for each cell cycle group. **(b)** Heatmaps show 𝛼, *β*, and *γ* estimated by cellDancer (first column) is associated with the experimentally derived synthesis and degradation status in scEU-seq (second column) in the cell cycle progress. **(c)** The phase portraits of cell cycle genes show the predicted kinetic parameters are related to experimental measurements in scEU-seq. **(d)** The velocities derived from cellDancer for metabolic labeling dataset are visualized on the relative position along the cell cycle using the Geminin-GFP and the Cdt1- RFP signals from the FUCCI system. Gene-shared pseudotime on the relative position is consistent with the experimental cell cycle position.

A previous study showed that metabolic labeling technology, such as sequencing mRNA labeled with 5-ethynyl-uridine (EU) in single cells (scEU-seq), can measure the synthesis and degradation of mRNA using the sequencing method^40^. Furthermore, Qiu et al.^22^ showed that scEU-seq can be used to predict the dynamics of the cell cycle. To investigate whether the predicted kinetic parameters are consistent with the experimental measurements, we used metabolic labeling data (that is, scEU-seq) of RPE1-FUCCI cells at specific points during cell cycle progression as a benchmark^40^. We first clustered RPE1-FUCCI cells into eight groups based on cell cycle stages and calculated the average spliced and unspliced expression of cell-cycle-associated genes, which also have synthesis and degradation rates in scEU-seq (Extended Data Fig. 5a). We applied cellDancer to predict the velocities and kinetic parameters of cell cycle genes and compared the predicted *α* and *γ* to the experimentally derived synthesis and degradation rates measured by scEU-seq^40^ (Extended Data Fig. 5b). Overall, the predicted *α* and *γ* are associated with the experimental measurements of mRNA synthesis and degradation (Extended Data Fig. 5b,c), especially in the highly expressed genes (Extended Data Fig. 5a). We also observed a difference between the predicted *α* and scEU-seq synthesis rates in the G1 state for the low-expression genes, of which expression starts to increase at the G1 state (Extended Data Fig. 5a). Our prediction captures this increase by a relatively large *α* in the G1 state, whereas scEU-seq shows a low synthesis rate, which may be due to the potential limitation of scEU-seq in the low-expression genes. Next, we predicted the velocity flow and pseudotime of the cell cycle procession using cell cycle genes. cellDancer predicts the direction of transcriptome shifting and the pseudotime during the cell cycle (Extended Data Fig. 5d). Together, the cellDancer-predicted kinetic parameters reflect the reality of mRNA turnover rates in cell cycle.

**Extended Data Fig. 6:**
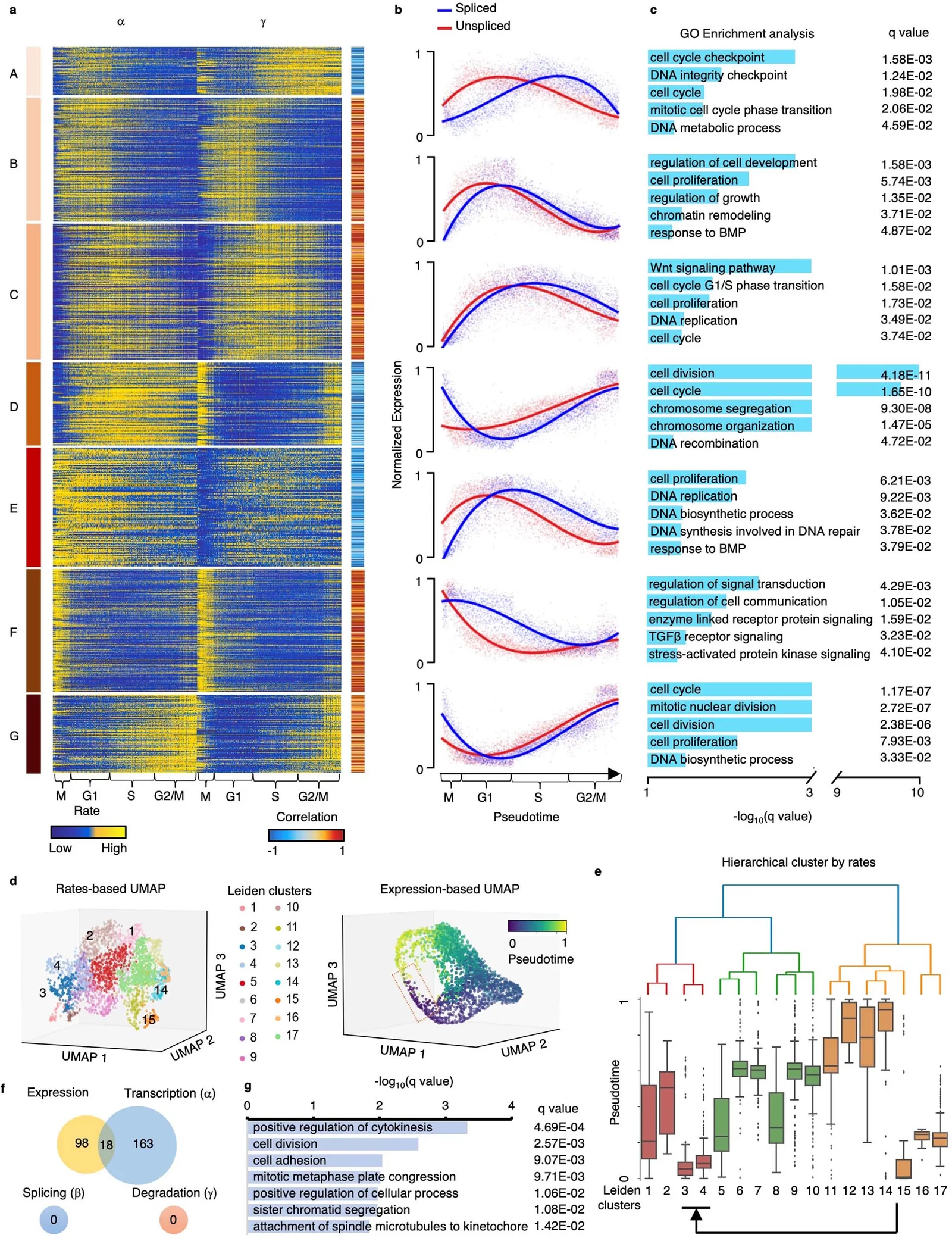
The dynamic pattern of rates identifies the different turnover strategies of genes, and cell-specific reaction rates reveal cell subpopulation uncaptured by expression. **(a)** 𝛼 and *γ* of genes along pseudotime. Genes are clustered into seven groups according to their dynamic patterns of 𝛼 and *γ*. The Pearson correlation coefficients between 𝛼 and *γ* are calculated. **(b)** The normalized spliced and unspliced reads of genes along pseudotime in each clustered group. **(c)** The GO pathway enrichment analysis using adjusted *p-*values of Fisher’s Exact test (Benjamini–Hochberg procedure, one-sided, *p* < 0.05) for genes in each group. **(d)** The 3D UMAP based on 𝛼, *β*, and *γ* colored by Leiden clusters (top) and the 3D UMAP based on expression colored by cell cycle pseudotime (bottom). **(e)** The hierarchical tree of the Leiden clusters. The box plot (n = 3,058 cells) shows the pseudotime of each cluster. Middle line in box plot, median; box boundary, interquartile range; whiskers, 10–90 percentile; minimum and maximum, not indicated in the box plot; gray dots, individual datapoints. **(f)** Venn diagram of genes with significant difference (*p* < 0.05, FC > 1.5 or FC < 1/1.5) on expression, 𝛼, *β*, and *γ* between the clusters 3 & 4 and other clusters. **(g)** The GO pathway enrichment analysis using adjusted *p-*values of Fisher’s Exact test (Benjamini–Hochberg procedure, one-sided, *p* < 0.05) of DAVID for the 163 genes that only differential in 𝛼.

We further investigated the functions of genes with different kinetic patterns. We grouped genes into seven clusters according to dynamic patterns of *α* and *γ* (Extended Data Fig. 6a). We calculated the correlation of *α* and *γ* and the average expression in each cluster (Extended Data Fig. 6b). We identified three positively correlated groups and four negatively correlated groups, indicating different turnover strategies in the clusters. Next, we investigated the functions of genes in each cluster through DAVID^21^ (Extended Data Fig. 6c). Overall, all clusters are associated with cell cycle pathways, including cell division, proliferation, chromatin remodeling, DNA replication and cell cycle checkpoints. We noticed that the genes in cluster F have large transcription and degradation rates in the mitosis stage, indicating a fast turnover of mRNAs. The genes in cluster F are enriched in pathways related to cell communication, including signal transduction, enzyme-linked receptor protein signaling, TGF-β receptor signaling and stress-activated protein kinase signaling, suggesting a quick communication of cells during mitosis.

To investigate the capacity of cell-specific rates in identifying cell subpopulations, we recaptured that pseudotime is continuous in the gene expression space during the cell cycle. Specifically, the G2 phase (pseudotime 0.8~1) is in proximity to the M phase (pseudotime 0~0.2) (Extended Data Fig. 6d). Then, we clustered the cells into 17 subpopulations according to the cell-specific rates (Extended Data Fig. 6d) using SCANPY^41^ and used the hierarchical method to further cluster each subpopulation (Extended Data Fig. 6e). We found that these subpopulations were globally clustered together in good agreement with cell cycle pseudotime except clusters 3 and 4 (a cell subpopulation at the M phase). The reaction rates of this cell subpopulation are more in line with clusters 1 and 2, which are at the G1 and S stages (Extended Data Fig. 6e). Next, we compared the gene expression and reaction rates of this intricate cell subpopulation with the other cells. We identified 116 differentially expressed genes and 181 genes having differential transcriptional rates by comparing this subpopulation to the rest and found that only 10% of genes having differential transcriptional rates were captured by the raw expression (Extended Data Fig. 6f). We further investigated the enriched pathways of these 163 genes that are uniquely identified by the rates through DAVID^21^. Those genes are enriched with cell division pathways, such as cytokinesis, cell division and mitotic metaphase congression (Extended Data Fig. 6g), suggesting that transcriptional regulation plays an important role in cell division at the M stage.

### Decoding human embryonic glutamatergic neurogenesis

**Extended Data Fig. 7:**
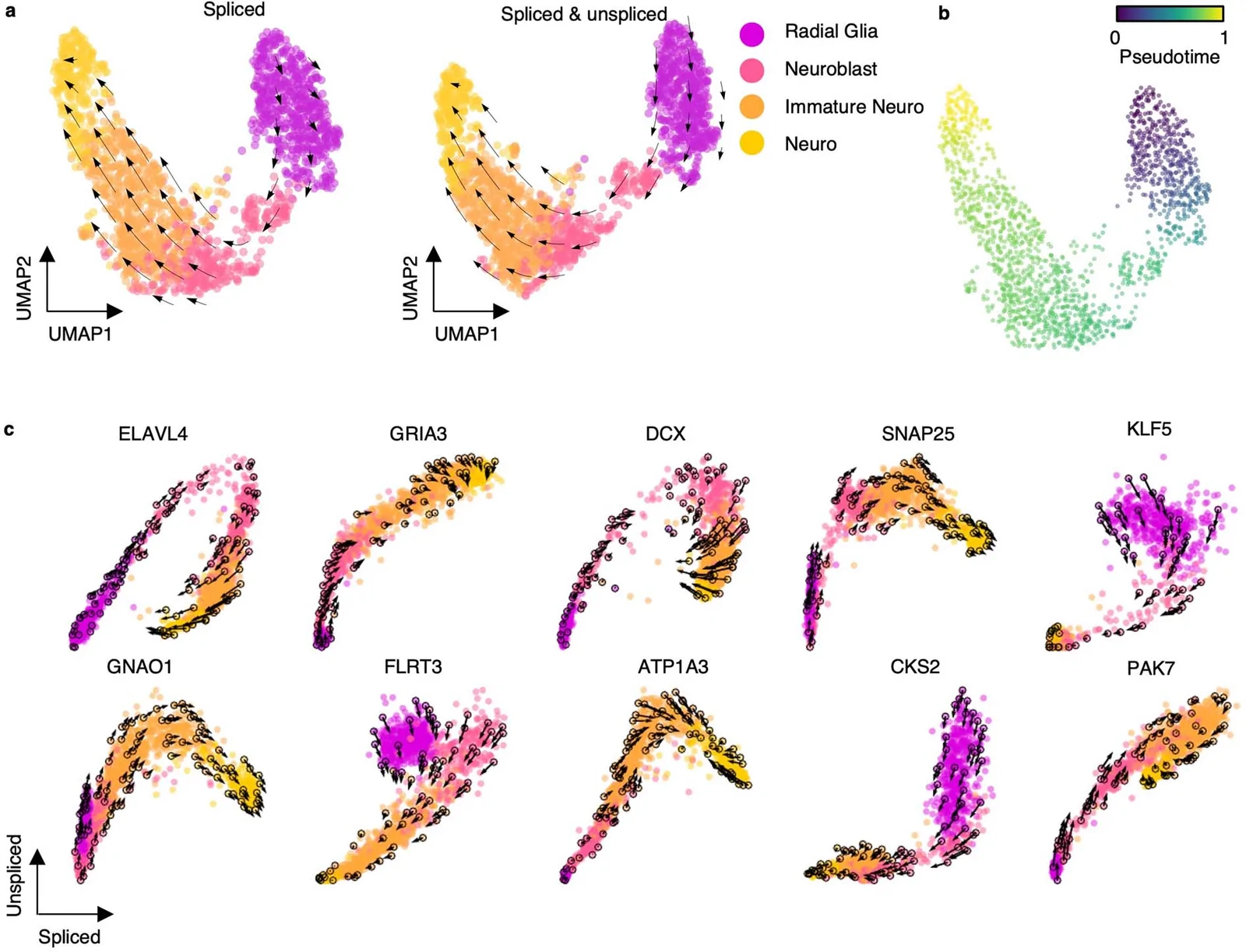
cellDancer decodes human embryonic glutamatergic neurogenesis. **(a)** The velocities derived from cellDancer for human embryo glutamatergic neurogenesis are visualized on the UMAP embedding based on spliced reads (left), and on the UMAP embedding based on the spliced and unspliced reads (right). **(b)** Gene-shared pseudotime projected on UMAP shows the order of cell development during neurogenesis. **(c)** RNA velocities predicted by cellDancer are projected onto the phase portraits for a set of selected genes.

We further investigated RNA velocity on an scRNA-seq dataset of the developing human forebrain at 10 weeks after conception, which was used as a benchmark in previous studies^12,42^. We used cellDancer to predict RNA velocity on human embryonic glutamatergic neurogenesis. The velocity on the embedding space and the derived pseudotime show that cellDancer accurately recaptures the cell fate of human embryonic glutamatergic neurogenesis (Extended Data Fig. 7a,b). The velocities of genes that are vital to neural development and neurogenesis, such as *ELAVL4* (ref. ^43^) and *DCX*^33,34^, were also correctly predicted (Extended Data Fig. 7c).

To test whether cellDancer is sensitive to the methods of neighbor cell detection, we applied cellDancer to predict velocity vector flow based on the nearest neighbors defined by the spliced RNAs or by the spliced and unspliced RNAs. Results suggest that the prediction of velocities using spliced RNAs is consistent with the prediction using spliced and unspliced RNAs (Extended Data Fig. 7a).

### cellDancer has a robust and efficient performance

**Extended Data Fig. 8:**
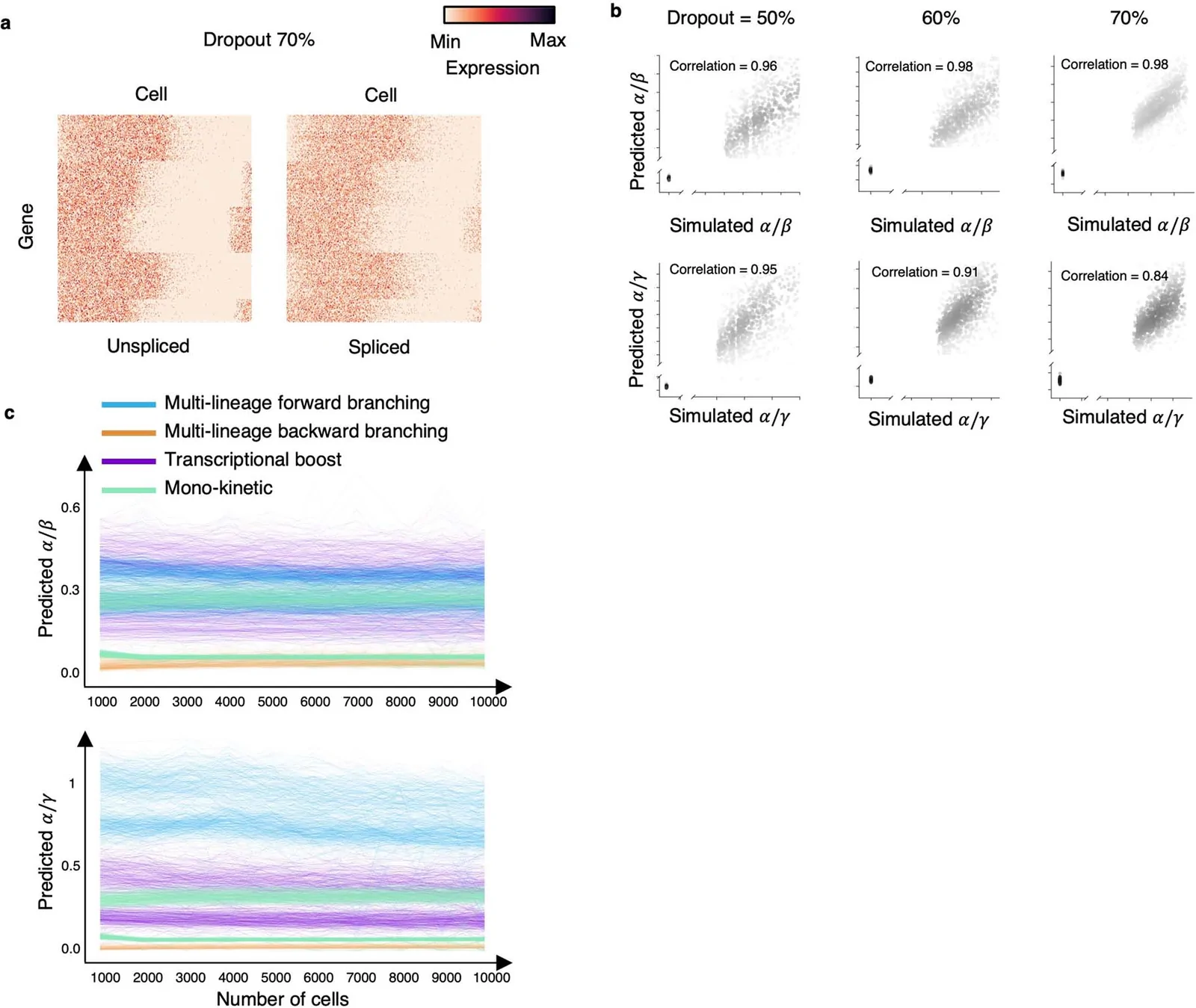
Robustness under different dropout ratios and number of cells. **(a)** Heatmaps show the overview of the simulated genes with a dropout of 70% on both unspliced and spliced reads. We simulated raw mRNA counts using a Poisson distribution to obtain the unspliced and spliced abundances with 50%, 60%, and 70% technical zeros. **(b)** Scatter plot shows a high correlation between the simulated (background truth) and the predicted 𝛼/𝛽 (top) and 𝛼/𝛾 (bottom) under different dropout ratios of the spliced and unspliced reads. The dropout was applied to both spliced and unspliced reads. The Pearson correlation coefficients between the prediction and the simulation are computed. The Pearson correlation coefficient in data with different dropout rates is larger than 0.96 and 0.84 for *α*/*β* and *α*/*γ*, respectively. **(c)** The predicted 𝛼/𝛽 (top) and 𝛼/𝛾 (bottom) are plotted against numbers of cells on the simulated mono-kinetic, multi-lineage forward branching, transcriptional boost, and multi-lineage backward branching genes.

The high proportion of zero reads is a key feature in scRNA-seq data, one cause of which is technical dropout. We tested whether cellDancer is robust with technical dropout (Extended Data Fig. 8a). cellDancer was able to correctly predict the gene dynamics even with high dropout ratios and learned RNA velocities in noisy scRNA-seq data (Extended Data Fig. 8b).

Next, we tested the robustness of our algorithm among different cell numbers. We gradually reduced the number of cells from 10,000 to 1,000 in the simulation dataset to predict RNA velocity and compared the prediction of *α*/*β* and *α*/*γ*. Results show that our model is robust in data with sparsity (Extended Data Fig. 8c).

**Extended Data Fig. 9:**
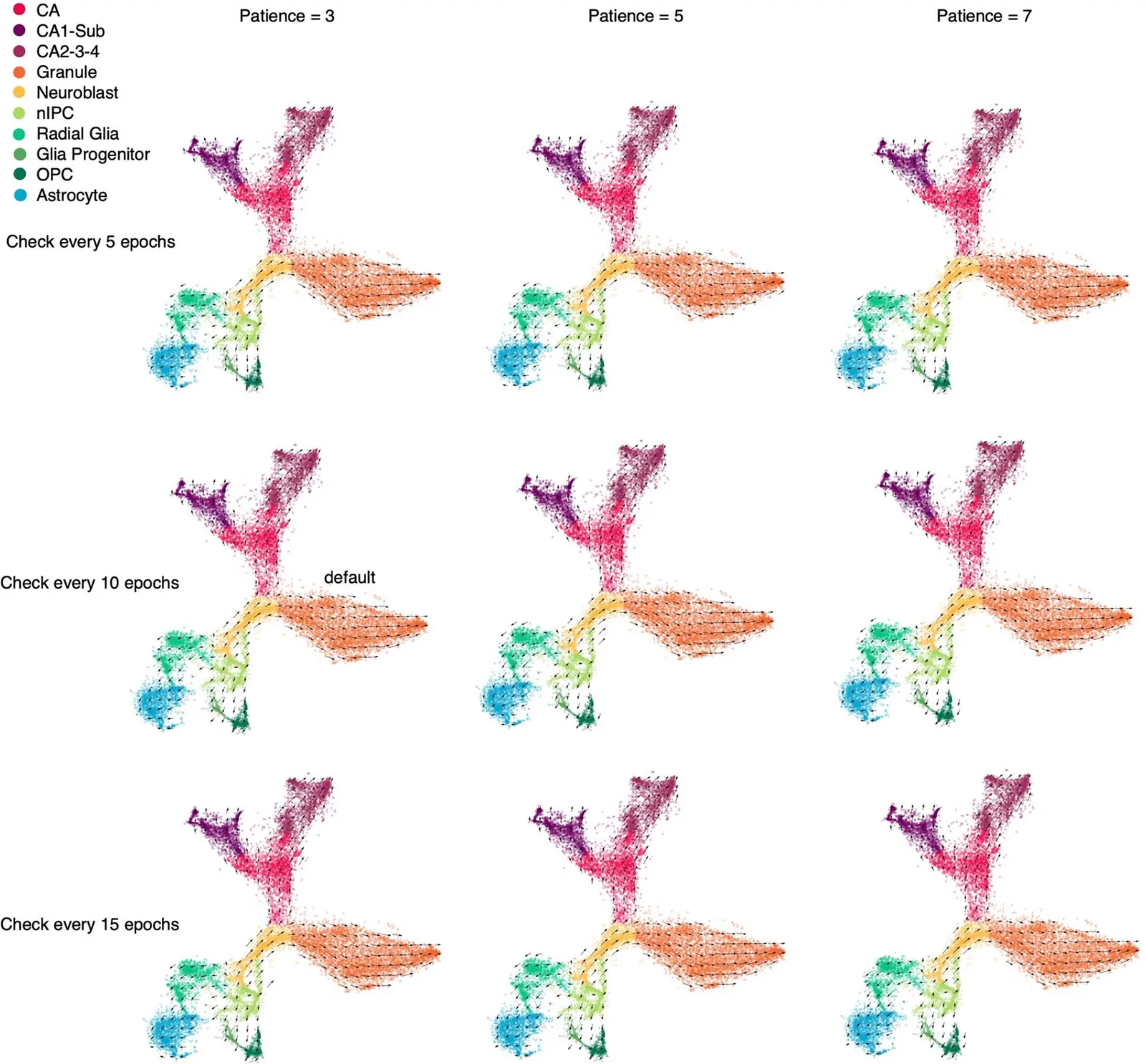
Sensitivity of the stopping criteria for the DNN training. The velocities derived by cellDancer with different combinations of stopping criteria parameters. The ‘check every n epoch’ means the number of epochs to skip (or a checkpoint) when computing the loss function. cellDancer calculates the loss of DNN every several epochs, which is specified by the checkpoint. The patience means the number of checkpoints waited before stopping the training when the loss score doesn’t decrease.

**Extended Data Fig. 10:**
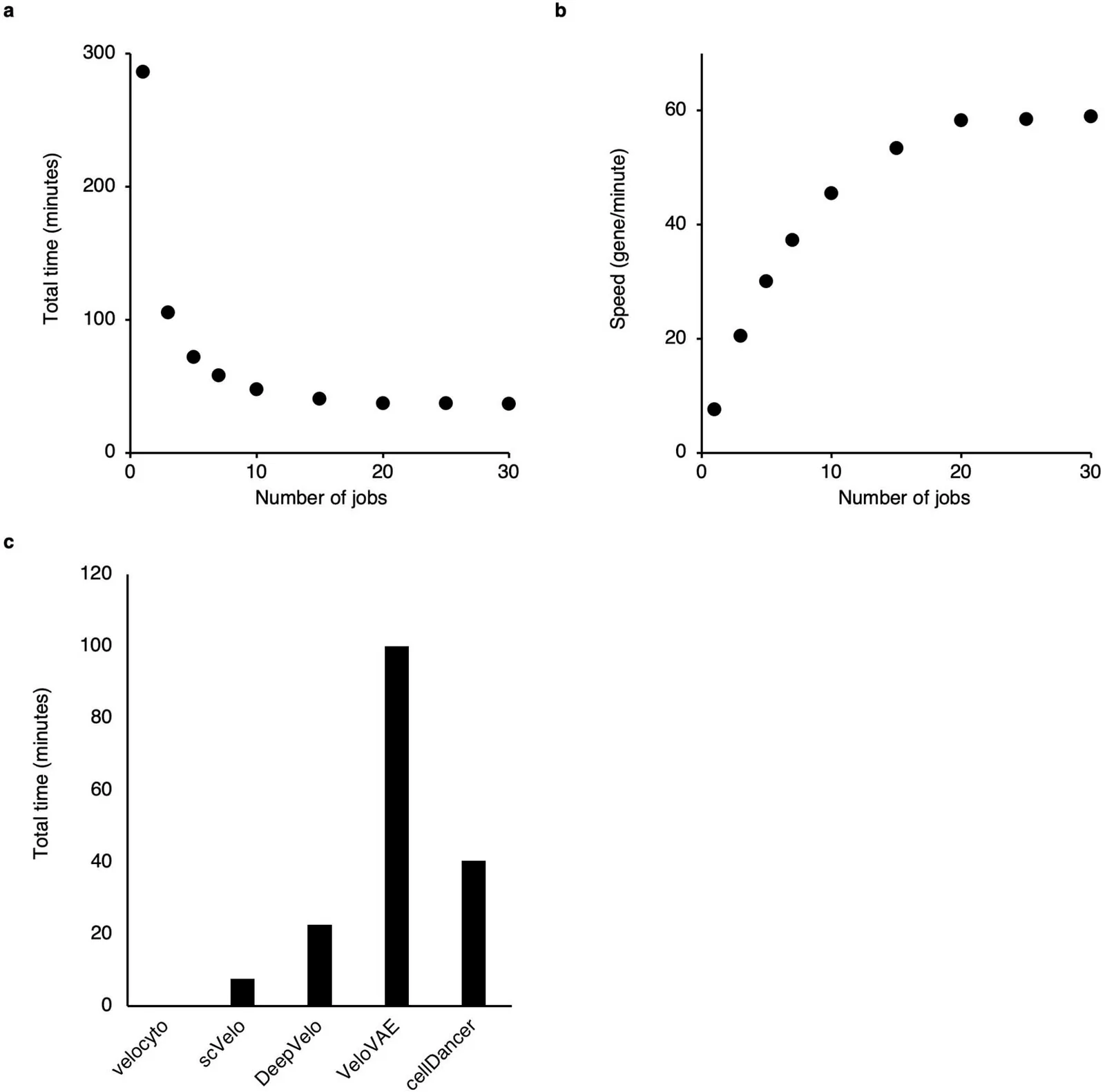
The speedup of cellDancer. (**a, b**) Scatter plots showing the total time **(a)** and the training speed **(b)** of cellDancer when applying multiprocessing. We tested the parallel speedup ratio of cellDancer by increasing job numbers from 1 to 30. We applied full analysis of cellDancer to 2,159 genes in 18,140 cells (the hippocampal dentate gyrus neurogenesis dataset) with the default parameters and calculated the runtime and speed of different job numbers. The evaluation of all the algorithms and the speedup ratio analysis was performed on a 2.7 GHz 24-Core Intel Xeon W processor. Total runtime decreases from 286 to 36 minutes when adding job numbers from 1 to 30 and reaches saturation at 15 jobs with 40 minutes. cellDancer has a feasible runtime of 53 genes per minute using 15 jobs. The training speed (number of genes per unit time) increases with the number of jobs. **(c)** Bar plot showing the total time of the comparison between velocyto, scVelo, DeepVelo, VeloVAE, and cellDancer. We compared the runtime of cellDancer with velocyto, scVelo, DeepVelo, and VeloVAE. The benchmark is based on 18,140 cells and 2,159 genes in the hippocampal dentate gyrus neurogenesis dataset with the default parameters. We set the number of jobs (threads) to 15 for scVelo, DeepVelo, VeloVAE, and cellDancer. cellDancer shows a comparable running time with the other two deep learning algorithms.

We tested the sensitivity of the stopping criteria for the training of cellDancer DNN. Two key parameters, ‘checkpoint’ and ‘patience’, are associated with the stopping criteria. We performed the full cellDancer analysis in the mouse hippocampus development experiment using a different number of checkpoints and patience for training. cellDancer shows low sensitivity to the stopping criteria of training (Extended Data Fig. 9). Furthermore, cellDancer independently predicted an individual DNN for each gene, which allows us to apply the multi-processing approach to speed up the efficiency. Overall, cellDancer has an optimized runtime (Extended Data Fig. 10).

## Discussion

In this study, we first showed that RNA velocity was automatically inferred from a neural network by optimizing a simple loss function based on local cosine similarity and implemented this deep learning algorithm to cellDancer, which is a flexible, robust and extensible framework for velocity inference. Our algorithm delivers four innovations. First, cellDancer overcomes the barriers for inferring RNA velocity with multiple kinetics, such as branching genes and transcriptional boost genes by local but not global velocity estimation. cellDancer also largely improves the reaction rates inference from bulk to single-cell resolution and illuminates the regulation of transcription, splicing and degradation at a single-cell resolution.

Second, cellDancer can be adapted to other velocity ordinary differential equations (ODEs) using the same framework. cellDancer does not require an analytic solution for ODEs. Therefore, cellDancer can be conveniently extended from original velocity ODEs to other extended ODEs. For example, scVelo and another recent study, UniTVelo^44^, proposed two stochastic models that considered the second-order moments of dynamics of the transcriptome to resolve cell-specific dynamics. To adapt to those velocity models, we could modify step 2 (computing predicted spliced/unspliced mRNA abundance) in the cellDancer workflow by using the velocity ODEs without changing other steps.

Third, cellDancer is highly modularized and extensible to multi-omics velocity models. As explained in the Methods, cellDancer is applicable to dynamics governed by first-order rate equations. More generally, in principle, cellDancer fits any dynamics following these rate kinetics:

$$\frac{{{\mathrm{d}}{{{\bf{T}}}}(t)}}{{\mathrm{d}}{t}} = f\left( {{{{\bf{T}}}}\left( t \right),{{{\bf{R}}}}\left( t \right)} \right)$$

where **T**(*t*) is the abundance vector of mRNAs, proteins, etc.; **R**(*t*) is the reaction rates vector; and *f* is a function of **T**(*t*) and **R**(*t*) and does not explicitly contain time *t*. For instance, Gorin et al.^45^ developed a protein velocity model by extending the RNA velocity model to cell surface protein translation. The protein velocity model has one more equation than the RNA velocity model to delineate the translation process. cellDancer can adapt to protein velocity by adding protein abundance into the input matrix and updating the module of loss function from RNA velocity to protein velocity. Moreover, chromatin accessibility measured by single-cell assay for transposase-accessible chromatin with sequencing (scATAC-seq)^46^ can be likewise included in cellDancer to reinforce the estimation of the transcription rates.

Finally, cellDancer DNN is scalable. A small, fully connected DNN was used in cellDancer to boost the running speed. If the relationship between kinetic parameters and spliced/unspliced mRNA abundance is complex, or multi-omics data are included in the velocity model, the fully connected DNN can be replaced or extended by other DNNs, such as a long short-term memory (LSTM) network^47^ or a convolutional neural network (CNN)^48^. This feature allows us to customize an optimal network structure based on the complexity of the velocity model and experimental data. Furthermore, due to the limitation that scRNA-seq captures only spliced and unspliced mRNA abundances, it is unfeasible to infer the absolute magnitude of the RNA velocity and the underlying (*α*,*β*,*γ*) values using only scRNA-seq data. Additional time information introduced by experimental techniques, such as metabolic labeling or different timepoint datasets, could be incorporated to obtain such absolute kinetic rates. This functionality would be included in a future version of cellDancer.

Together, cellDancer represents a notable advance to quantitatively predict the time evolution of cellular transcriptomics, applicable to numerous biological models and disease processes at a genome-wide scale.

## Methods

### Modeling RNA transcriptional dynamics

The reaction kinetics of a single gene is described by two ordinary differential equations:

1 $$\frac{{{\mathrm{d}}u\left( t \right)}}{{\mathrm{d}t}} = \alpha \left( t \right) - \beta \left( t \right)u\left( t \right)$$

2 $$\frac{{{\mathrm{d}}s\left( t \right)}}{{\mathrm{d}t}} = \beta \left( t \right)u\left( t \right) - \gamma \left( t \right)s\left( t \right)$$

where *u*(*t*) and *s*(*t*) are time-dependent concentrations of the premature and mature mRNAs, and *α*, *β*, *γ* indicate the transcription, splicing and degradation rates, respectively. For simplicity, one of the key assumptions in previous models for estimation of RNA velocity is that *α* is either a constant (velocyto model) or a binary (scVelo model) value, and *β* and *γ* are shared by all the genes and cells. However, the assumption fails in evaluation of a heterogeneous cell subpopulation. In this study, we developed cellDancer, a deep learning framework, to generalize estimation of RNA velocity in both homogeneous and heterogeneous cell populations by predicting cell-specific time-dependent *α*, *β* and *γ* from premature and mature reads. A unique feature of the cellDancer framework is its capability to determine gene-specific kinetics that can be described by the rate equations (Eqs. 1 and 2).

In cellDancer, we use a DNN with a set of network parameters (*θ*) to learn the unknown functions that map the predictive features to the rates. Specifically, for gene *i* in the scRNA-seq dataset, there are *n* captured cell snapshots (*t* = *t*_1_, *t*_2_,…,*t*_*n*_) at different stages of the cell development (for simplicity, we also refer to time *t*_*j*_ as ‘cell *j*’ throughout the paper), and we formulated the reaction rates as functions of the abundances of the unspliced and spliced mRNAs in Eq. 3:

3 $$\left( {\alpha ^i(t),\beta ^i(t),\gamma ^i(t)} \right)^{{{\mathrm{T}}}} = {{\Phi }}_{\theta ^i}\left( {u^i\left( t \right),s^i\left( t \right)} \right)$$

where the DNN is described as a mapping Φ with gene-specific network parameters (*θ*^*i*^). To train the DNN, we send one gene to the DNN at a time. We randomly sample a subset of cells (details in the ‘Model parameters’ subsection) as the input in each epoch of training. We leave out the superscript notation *i* in the following detailed steps for prediction.

First, the reaction kinetics ODEs in Eqs. 1 and 2 are discretized:

4 $$\frac{{u\left( {t + \Delta t} \right) - u\left( t \right)}}{{\Delta t}} = \alpha \left( {u\left( t \right),s\left( t \right)} \right) - \beta \left( {u\left( t \right),s\left( t \right)} \right)u\left( t \right),$$

5 $$\frac{{s\left( {t + \Delta t} \right) - s\left( t \right)}}{{\Delta t}} = \beta \left( {u\left( t \right),s\left( t \right)} \right)u\left( t \right) - \gamma \left( {u\left( t \right),s\left( t \right)} \right)s\left( t \right),$$

where pseudotime *t* is discretized and Δ*t* is an infinitesimal time increment. We use cellDancer to jointly predict cell-specific *α*(*u*(*t*_*j*_), *s*(*t*_*j*_)), *β*(*u*(*t*_*j*_), *s*(*t*_*j*_)) and *γ*(*u*(*t*_*j*_), *s*(*t*_*j*_)) given spliced and unspliced mRNA abundance *u*(*t*_*j*_) and *s*(*t*_*j*_) of cell *j*. Second, we use the predicted rates to calculate the extrapolated mRNA abundance *s*(*t*_*j*_ + Δ*t*) and *u*(*t*_*j*_ + Δ*t*) by the discretized reaction kinetics. To measure the difference between predicted and true velocity vectors, we define a loss function ${{{\mathcal{L}}}}$ based on every cell’s cosine similarity between the predicted and observed velocity vectors:

6 $${{{\mathcal{L}}}} = \mathop {\sum}\limits_{j = 1}^n {{{{\mathcal{L}}}}_j} ,$$

7 $${{{\mathcal{L}}}}_j = 1 - \mathop {{\max }}\limits_{\{\, j^\prime \} } \frac{{v_j \cdot v_j^\prime }}{{\left| {v_j} \right| \ast \left| {v_j^\prime } \right|}},$$

8 $$v_j = \left( {u\left( {t_j + \Delta t} \right) - u\left( {t_j} \right),s\left( {t_j + \Delta t} \right) - s\left( {t_j} \right)} \right),$$

9 $$v_j^\prime = \left( {u\left( {t_{j^\prime }} \right) - u\left( {t_j} \right),s\left( {t_{j\prime }} \right) - s\left( {t_j} \right)} \right),$$

${{{\mathcal{L}}}}$ (${{{\mathcal{L}}}}_j$) is the overall (cell *j*) loss function; *v*_*j*_ ($v_j^\prime$) is the predicted (observed) RNA velocity vector, where {*j*′} is a collection of cells in the neighborhood of cell *j*; and $t_{j^\prime }$ is the observed cell in the neighboring cells {*j*′} that minimizes the loss function for cell *j*. Note that the neighboring cells are controlled by the number of n_neighbors and can be either gene-specific (calculated in the phase space of each gene) or gene-shared (calculated in the embedding space using the abundances of the spliced mRNA or the abundances of both the spliced and the unspliced mRNA).

Finally, we obtain *θ*^*i*^ by minimizing the overall loss function ${{{\mathcal{L}}}}$ for gene *i* by applying the Adam optimization algorithm in a DNN. The configuration of the DNN is as follows: an input layer with 2*n* nodes; two fully connected hidden layers each with 100 nodes and the leaky ReLU activation function; and an output layer with 3*n* nodes. The sigmoid activation function $\sigma \left( x \right) = \frac{1}{{1 + e^{ - x}}}$ is applied as a regularization to constrain the outputs (*α*, *β* and *γ*) within the range [0, 1]. The learning rate of the Adam optimizer is 0.001. The weight decay is 0.004, which adds L2 penalty to the weights parameters and prevents overfitting. The training of the DNNs is terminated if the loss function does not decrease after three checkpoints. Those training parameters are fully controllable by the user in the cellDancer command line interface. The DNN in cellDancer is implemented using PyTorch Lightning^49^, a widely used Python library.

### Simulation details

To assess the accuracy and limitation of cellDancer, we generate various kinetic regimes of the expression profiles using time-dependent rates of transcription, splicing and degradation (*α*,*β*,*γ*). Specifically, for one gene, a set of differential equations is solved by numerical integration using the function *integrate.solve_ivp* under the SciPy package^50^ with the Runge–Kutta method^51,52^. The unspliced and spliced reads are initialized to 0. Gaussian noises are added to the generated gene expression level for each cell.

We simulate the spliced and unspliced expression of 2,000 cells and 1,000 genes for transcriptional boost, multi-forward branching and multi-backward branching regimes. For transcriptional boost genes, *α* is sampled from a uniform distribution of *U*(1.6, 2.4) before boosting and *U*(4, 6) for cells after boosting where the lower and upper limits are set by varying 20% from the mean values of 2 (before boosting) and 5 (after boosting). *β* is sampled from a uniform distribution of *U*(1.8, 2.2) for all cells where the lower and upper limits are set by varying 10% from the mean value of 2. *γ* is sampled from a uniform distribution of *U*(0.9, 1.1) where the lower and upper limits are set by varying 10% from the mean value of 1 for all cells. For multi-forward branching genes, *α* is sampled from a uniform distribution of *U*(0.8, 1.2) for cells in the first lineage and *U*(4, 6) for cells in the second lineage where the lower and upper limits are set by varying 20% from the mean values of 1 (first lineage) and 5 (second lineage). *β* is sampled from a uniform distribution of *U*(0.4, 0.6) for cells in the first lineage and *U*(0.8, 1.2) for cells in the second lineage where the lower and upper limits are set by varying 20% from the mean value of 0.5 (first lineage) and 1 (second lineage). *γ* is sampled from a uniform distribution of *U*(0.2, 0.3) for cells in the first lineage and *U*(4, 6) for cells in the second lineage where the lower and upper limits are set by varying 20% from the mean values of 0.25 (first lineage) and 5 (second lineage). For multi-backward branching genes, *α* is set to 0 in all cells. *β* and *γ* are sampled from a uniform distribution of *U*(0.9, 1.1) where the lower and upper limits are set by varying 10% from the mean value of 1 for all cells. In the first lineage, cells start from a region around a point of (*s* = 1.3, *u* = 0.2) to decrease. In the second lineage, cells start from a region around a point of (*s* = 1, *u* = 1) to decrease. The data are used as input of a standard cellDancer analysis pipeline. After velocity estimation, we calculate an error rate to evaluate the accuracy of cellDancer against the ground truth velocity. The error rate is calculated as the percentage of cells having a low correlation coefficient (lower than 0.7 as a cutoff) between the estimated velocity and the ground truth velocity.

To investigate the robustness of cellDancer in data with high technical dropout, we simulate dropout in the expression of unspliced and spliced mRNAs. According to the experimental datasets in this study, the average dropout ratios for the unspliced and spliced mRNA reads are in the range of 50% to 70% for the top 2,000 highly variable genes. Therefore, for dropout ratios of 50%, 60% and 70%, we simulate 1,000 genes each. To achieve this, we first generate the spliced and unspliced abundances ($u_j^i$ and $s_j^i$ for gene *i* of cell *j*), which follow the transcriptional dynamics equations (Eqs. 1 and 2). We assume that those abundances are averaged over the raw counts ($U_{\{ j^\prime \} }^i$ and $S_{\{ j^\prime \} }^i$) of the neighboring cells, as in real scRNA-seq data those raw counts are zero-inflated. Based on this assumption, for a gene *i* in any given cell *j*, we randomly generate spliced and unspliced raw counts that follow the Poisson law $\left(U_{\{ j^\prime \} }^i\sim {\mathrm{Poisson}}\left( {u_j^i} \right)\right.$ and $\left.S_{\{ j^\prime \} }^i\sim {\mathrm{Poisson}}\left( {s_j^i} \right)\right)$ for 200 neighboring cells {*j*′}. We perform a grid search for the kinetic rate parameters (*α*,*β*,*γ*) in the range [0.1, 1.0] at a step of 0.1. We use kinetic parameters that lead to dropout ratios (50% ± 3%, 60% ± 3% and 70% ± 3%) in our RNA velocity estimation, where the averaged raw counts (sample average) are used for the unspliced and spliced abundances.

### Pseudotime estimation

The RNA velocity vector for a cell *j* is represented by a high-dimensional vector $v_j = \left( {v_j^1,v_j^2, \ldots ,v_j^g} \right)$, where *g* is the total number of genes and $v_j^i$ is the velocity for gene *i* in cell *j*. Following the method of velocyto and scVelo, we project the velocity vectors of the cells into the low-dimensional embedding space {*ξ*}_*dim*_ using embedding algorithms such as PCA, t-distributed stochastic neighbor embedding (t-SNE) or UMAP for visualization and gene-shared pseudotime estimation. Under the assumption that the more correlated the change in the gene expression $\delta _{jj^\prime } = s_j - s_j^\prime$ from cells *j* and *j*′ with the direction of the velocity *v*_*j*_, the higher chance that cell *j* could transition to cell *j*′, we construct the transition probability matrix by applying an exponential kernel to the correlation between $\delta _{jj^\prime }$ and *v*_*j*_:

10 $$P_{jj^\prime } \propto e^{\frac{{corr\left( {v_j,\delta _{jj^\prime }} \right)}}{\sigma }},$$

where *σ* = 0.05. A normalization factor is applied to ensure the sum of transition probabilities for cell *j* to its neighboring cells (*N*, which is determined by *k*-nearest neighbors in the high-dimensional space or optionally the low-dimensional embedding space) is 1:

11 $$\mathop {\sum}\nolimits_{j^\prime \in N} {P_{jj^{\prime} }} = 1,$$

The velocity of cell *j* on the low-dimensional embedding space {*ξ*} is estimated as

12 $$\tilde v_j = \mathop {\sum}\nolimits_{j^\prime \in N} {\left( {P_{jj^{\prime} } - 1} \right)} {\hat{\theta}}_{jj^{\prime} },$$

where ${\hat{\theta}} _{jj^{\prime} }$ is the unitary vector of the displacement between cell *j* and *j*′ in the embedding space.

To detect the cell state transition paths and track the continuous changes in transcriptome along those paths, we sort the cells in temporal order by carrying out cell (gene-shared) pseudotime analysis based on the RNA velocities. First, we divide the low-dimensional embedding space {*ξ*} to a customized grid to smooth the abrupt velocity vector flows, and the velocity of a cell *j* in a grid *I* (or ‘meta cell’) is estimated as the mean velocity ${\tilde{v}}_I$ of the enclosed cells. We then generate a pool of trajectories $\left\{ {\xi _j^r\left( {t_0} \right),\xi _j^r\left( {t_1} \right),\xi _j^r\left( {t_2} \right), \ldots } \right\}_{j = 1, \ldots ,n_{{{{\mathrm{cells}}}}}}^{r = 1, \ldots ,n_{{{{\mathrm{repeats}}}}}}$ tracing the velocity streamlines starting from any cell *j* using the following equation of motion:

13 $$\xi _j\left( {t + \Delta t} \right) = \xi _j\left( t \right) + {\tilde{v}}_I\Delta t.$$

A Gaussian-distributed swaying angle *θ* ∈ *N*(0, *π*/6) is applied at every timestep to allow a slight deviation from the direction of the velocity flow. Second, from the trajectory pool, we select *m* trajectories {*L*_*k*_(*t*)}_*k* = 1,...,*m*_ whose traverse length is a local maximum (or long trajectories, as shown in Extended Data Fig. 2d for the erythroid maturation dataset). The traverse length is computed as the accumulated distance of a trajectory $\mathop {\sum}\nolimits_t \parallel \xi \left( {t + \Delta t} \right) - \xi \left( t \right)\parallel$. The long trajectories are determined by iteratively selecting the longest trajectory and eliminating its similar trajectories within a cutoff until no trajectory is left in the pool. The fate of a neighboring cell *j* is decided by whether most of the trajectories originated from the position of cell *j*, *ξ*_*j*_(*t*_0_), terminate on/around a long trajectory *L*_*l*_(*t*). The pseudotime *t*_*j*_ of cell *j* is then assigned as the time on *L*_*l*_(*t*), where *L*_*l*_(*t*_*j*_) is closest to *ξ*_*j*_(*t*_0_) (Extended Data Fig. 2d). Finally, at this moment, all the cells are assigned a relative time according to the respective paths, or ‘time zones’, and we need to adjust the relative time of the cells by finding the time shift between those ‘time zones’. This is done based on an assumption that ‘overlapping’ cells (in practice, we consider cells in close proximity) in the embedding space (or optionally in the high-dimensional expression space) also share the same time. The assumption is consistent with the assumption on which the transition probability matrix is based. The time for the cells in each ‘time zone’ (or cluster) is adjusted using a graph-based approach. The time adjustment algorithm is outlined below.

1. Construct the graph. Every cluster forms a node, and an edge is formed between nodes *l* and *m* if there is a time shift Δ*t*_*lm*_ = *t*_*l*_ − *t*_*m*_ between the ‘overlapping’ cells going for path *L*_*l*_ and path *L*_*m*_. Therefore, each cell abiding by the *L*_*m*_ ‘time zone’ needs addition of Δ*t*_*lm*_ to the original cell time to consolidate all the cell time in the two clusters.
2. Divide the graph into individual trees. If the graph is a forest, divide it into trees. If a cycle exists, the time adjustment algorithm fails. In the latter scenario, we suggest reducing the *n*_path parameter to reduce the number of long trajectories.
3. Compute the accumulative time shift $\tau _k^{{{{\mathrm{abs}}}}}$ needed for each node *k* ∈ {1,2,…, *n*_nodes_} in each tree *T* in a few steps.
4. Initiate $\{ \tau _k^{\mathrm{{abs}}}\}$ with 0 for each node *k* ∈ {1,2,…,*n*_nodes_}. Initiate a marker for each node {flag_*k*_} with 0.
5. Start from a node *o* and set the marker to 1. Traverse all the connections. For a connection between node *l* and *m*: add $\tau _m^{\mathrm{{abs}}}$ by Δ*t*_*lm*_ if *l* equals *o* and set the marker for node *m* to 1; subtract $\tau _l^{\mathrm{abs}}$ by Δ*t*_*lm*_ if *m* equals *o* and set the marker for node *l* to flag_*k*_ = 1. Repeat the process until all the nodes are marked as 1.

### scRNA-seq data and pre-processing

All scRNA-seq data in this study were downloaded publicly (see details in the ‘Data availability’ section).

1. For the pancreatic endocrinogenesis data, we followed the method by Bergen et al. in the scVelo study^17^ and filtered 3,696 cells with 2,000 genes for further analysis.
2. For the mouse hippocampal dentate gyrus neurogenesis data, we followed the gene and cell filtering methods by La Manno et al.^12^ and selected 18,140 cells with 2,159 genes.
3. For the erythroid lineage of the mouse gastrulation data, we selected 12,329 cells from cell types, including hematoendothelial progenitors, blood progenitors 1/2 and erythroid 1/2/3 in stages of E7.0, E7.25, E7.5, E7.75, E8.0, E8.25 and E8.5. We followed the standard data pre-processing procedures in scVelo with default parameters except that we used 100 nearest neighbors for the calculation of the first moment to reduce the noise in transcripts.
4. For the human embryo glutamatergic neurogenesis dataset, we kept cells with at least 200 genes expressed and kept genes that were captured in at least three cells. We identified all the high variable genes with the default settings of scanpy.pp.highly_variable_genes() by using SCANPY^41^. In total, 1,054 cells with 1,720 genes were selected. We used 200 nearest neighbors for the calculation of the first moment to reduce the noise in transcripts.
5. For the cell cycle progression in the REP1-FUCCI cells, we used the scEU-seq data, in which we took 3,058 cells with the top 2,000 high variable genes from the pulse experiment. The unspliced mRNA reads were calculated as the addition of the unspliced labeled and unspliced unlabeled reads, likewise for the spliced mRNA reads. We used 300 nearest neighbors for the calculation of the first moment to reduce the noise in transcripts. The synthesis and degradation rates (molecules per hour) measured by scEU-seq data were obtained from the study of the original paper^40^.

### Model parameters

In DNN training, the learning rate and patience are associated with the total number of training epochs. In all case studies, the learning rate was set to 0.001, which is widely used in Adam optimizer. The patience was set to 3 in all case studies. The time increment Δ*t* in Eqs. 4 and 5 was set to 0.5. The permutation ratio determines how many cells were sent to train the model in each epoch. We recommend using a large permutation ratio for datasets with a small number of cells or datasets presenting a clear pattern in gene phase portraits. Specifically, for gastrulation erythroid maturation (12,329 cells) and the cell cycle progression in REP1-FUCCI data (3,058 cells), we used the default permutation ratio of 0.125; for the mouse hippocampus development dataset (18,140 cells), we set the permutation ratio to 0.1; for the pancreatic endocrinogenesis data (3,696 cells), we set the permutation ratio to 0.5; and for the human embryo glutamatergic neurogenesis data (1,720 cells), we set the permutation ratio to 0.3. For all genes within the same dataset, the DNN parameters were kept the same.

### Reporting summary

Further information on research design is available in the Nature Portfolio Reporting Summary linked to this article.

## Online content

Any methods, additional references, Nature Portfolio reporting summaries, source data, extended data, supplementary information, acknowledgements, peer review information; details of author contributions and competing interests; and statements of data and code availability are available at https://doi.org/10.1038/s41587-023-01728-5.

## Supplementary information


Supplementary InformationSupplementary Figs. 1–3, Note 1 and Table 1.
Reporting Summary


## Data Availability

All the scRNA-seq raw data are publicly accessible. The pancreatic endocrinogenesis data can be extracted using scVelo’s CLI: *scvelo.datasets.pancreas()* or accessed from the original work^36^ under accession number GSE132188 of the Gene Expression Omnibus (GEO). The hippocampal dentate gyrus neurogenesis data can be accessed at http://pklab.med.harvard.edu/velocyto/DentateGyrus/DentateGyrus.loom or the original paper^53^ under GEO accession number GSE95753. The erythroid lineage of mouse gastrulation data can be extracted using scVeloʼs CLI: *scvelo.datasets.gastrulation()* or from the original work^2^ under accession number E-MTAB-6967 of ArrayExpress. Human embryo glutamatergic neurogenesis can be accessed at https://github.com/pachterlab/GFCP_2022/blob/main/notebooks/data/hgForebrainGlut.loom or the original work^12^ under Sequence Read Archive accession code SRP129388. Cell cycle progression in REP1-FUCCI cells can be extracted using dynamo’s CLI: *dyn.sample_data.scEU_seq_rpe1()* or from the original work^22^ under GEO accession number GSE128365.

## Code availability

cellDancer is implemented in Python and is available at https://github.com/GuangyuWangLab2021/cellDancer.

## References

[1] Qiu, C. et al. Systematic reconstruction of cellular trajectories across mouse embryogenesis. *Nat. Genet.* **54**, 328–341 (2022). 35288709 10.1038/s41588-022-01018-x PMC8920898

[2] Pijuan-Sala, B. et al. A single-cell molecular map of mouse gastrulation and early organogenesis. *Nature* **566**, 490–495 (2019). 30787436 10.1038/s41586-019-0933-9 PMC6522369

[3] Cao, J. et al. The single-cell transcriptional landscape of mammalian organogenesis. *Nature* **566**, 496–502 (2019). 30787437 10.1038/s41586-019-0969-x PMC6434952

[4] Cheng, S. et al. Single-cell RNA-seq reveals cellular heterogeneity of pluripotency transition and X chromosome dynamics during early mouse development. *Cell Rep.* **26**, 2593–2607 (2019). 30840884 10.1016/j.celrep.2019.02.031

[5] Mohammed, H. et al. Single-cell landscape of transcriptional heterogeneity and cell fate decisions during mouse early gastrulation. *Cell Rep.* **20**, 1215–1228 (2017). 28768204 10.1016/j.celrep.2017.07.009 PMC5554778

[6] Kharchenko, P. V. The triumphs and limitations of computational methods for scRNA-seq. *Nat. Methods* **18**, 723–732 (2021). 34155396 10.1038/s41592-021-01171-x

[7] Stephenson, E. et al. Single-cell multi-omics analysis of the immune response in COVID-19. *Nat. Med.* **27**, 904–916 (2021). 33879890 10.1038/s41591-021-01329-2 PMC8121667

[8] Kolodziejczyk, A. A., Kim, J. K., Svensson, V., Marioni, J. C. & Teichmann, S. A. The technology and biology of single-cell RNA sequencing. *Mol. Cell* **58**, 610–620 (2015). 26000846 10.1016/j.molcel.2015.04.005

[9] Setty, M. et al. Characterization of cell fate probabilities in single-cell data with Palantir. *Nat. Biotechnol.* **37**, 451–460 (2019). 30899105 10.1038/s41587-019-0068-4 PMC7549125

[10] Bendall, S. C. et al. Single-cell trajectory detection uncovers progression and regulatory coordination in human B cell development. *Cell* **157**, 714–725 (2014). 24766814 10.1016/j.cell.2014.04.005 PMC4045247

[11] Haghverdi, L., Buttner, M., Wolf, F. A., Buettner, F. & Theis, F. J. Diffusion pseudotime robustly reconstructs lineage branching. *Nat. Methods* **13**, 845–848 (2016). 27571553 10.1038/nmeth.3971

[12] La Manno, G. et al. RNA velocity of single cells. *Nature* **560**, 494–498 (2018). 30089906 10.1038/s41586-018-0414-6 PMC6130801

[13] Bergen, V., Soldatov, R. A., Kharchenko, P. V. & Theis, F. J. RNA velocity–current challenges and future perspectives. *Mol. Syst. Biol.* **17**, e10282 (2021). 34435732 10.15252/msb.202110282 PMC8388041

[14] Couturier, C. P. et al. Single-cell RNA-seq reveals that glioblastoma recapitulates a normal neurodevelopmental hierarchy. *Nat. Commun.* **11**, 3406 (2020). 32641768 10.1038/s41467-020-17186-5 PMC7343844

[15] Guerrero-Juarez, C. F. et al. Single-cell analysis of human basal cell carcinoma reveals novel regulators of tumor growth and the tumor microenvironment. *Sci. Adv.* **8**, 7981 (2022). 10.1126/sciadv.abm7981 PMC9187229 35687691

[16] Lederer, A. R. & La Manno, G. The emergence and promise of single-cell temporal-omics approaches. *Curr. Opin. Biotechnol.* **63**, 70–78 (2020). 31918114 10.1016/j.copbio.2019.12.005

[17] Bergen, V., Lange, M., Peidli, S., Wolf, F. A. & Theis, F. J. Generalizing RNA velocity to transient cell states through dynamical modeling. *Nat. Biotechnol.* **38**, 1408–1414 (2020). 32747759 10.1038/s41587-020-0591-3

[18] Barile, M. et al. Coordinated changes in gene expression kinetics underlie both mouse and human erythroid maturation. *Genome Biol.* **22**, 197 (2021). 34225769 10.1186/s13059-021-02414-y PMC8258993

[19] Cui, H., Maan, H. & Wang, B. DeepVelo: deep learning extends RNA velocity to multi-lineage systems with cell-specific kinetics. Preprint at https://www.biorxiv.org/content/10.1101/2022.04.03.486877v2 (2022). 10.1186/s13059-023-03148-9 PMC10799431 38243313

[20] Gu, Y., Blaauw, D. & Welch, J. D. Bayesian inference of RNA velocity from multi-lineage single-cell data. Preprint at https://www.biorxiv.org/content/10.1101/2022.07.08.499381v1 (2022).

[21] Huang, D. W. et al. DAVID Bioinformatics Resources: expanded annotation database and novel algorithms to better extract biology from large gene lists. *Nucleic Acids Res.* **35**, W169–W175 (2007). 17576678 10.1093/nar/gkm415 PMC1933169

[22] Qiu, X. et al. Mapping transcriptomic vector fields of single cells. *Cell* **185**, 690–711 (2022). 35108499 10.1016/j.cell.2021.12.045 PMC9332140

[23] Eich, C. et al. In vivo single cell analysis reveals Gata2 dynamics in cells transitioning to hematopoietic fate. *J. Exp. Med.* **215**, 233–248 (2018). 29217535 10.1084/jem.20170807 PMC5748852

[24] Lau, E. O.-C. et al. DIAPH3 deficiency links microtubules to mitotic errors, defective neurogenesis, and brain dysfunction. *eLife* **10**, e61974 (2021). 33899739 10.7554/eLife.61974 PMC8102060

[25] Laub, F. et al. Transcription factor KLF7 is important for neuronal morphogenesis in selected regions of the nervous system. *Mol. Cell. Biol.* **25**, 5699–5711 (2005). 15964824 10.1128/MCB.25.13.5699-5711.2005 PMC1157008

[26] Upadhyay, A. et al. Neurocalcin delta knockout impairs adult neurogenesis whereas half reduction is not pathological. *Front. Mol. Neurosci.* **12**, 19 (2019). 30853885 10.3389/fnmol.2019.00019 PMC6396726

[27] Yamagata, M., Duan, X. & Sanes, J. R. Cadherins interact with synaptic organizers to promote synaptic differentiation. *Front. Mol. Neurosci.* **11**, 142 (2018). 29760652 10.3389/fnmol.2018.00142 PMC5936767

[28] Michibata, H. et al. Inhibition of mouse GPM6A expression leads to decreased differentiation of neurons derived from mouse embryonic stem cells. *Stem Cells Dev.* **17**, 641–651 (2008). 18522499 10.1089/scd.2008.0088

[29] Feng, H., Khalil, S., Neubig, R. R. & Sidiropoulos, C. A mechanistic review on *GNAO1*-associated movement disorder. *Neurobiol. Dis.* **116**, 131–141 (2018). 29758257 10.1016/j.nbd.2018.05.005

[30] Grant, S. G. Synaptopathies: diseases of the synaptome. *Curr. Opin. Neurobiol.* **22**, 522–529 (2012). 22409856 10.1016/j.conb.2012.02.002

[31] Bartkowska, K., Paquin, A., Gauthier, A. S., Kaplan, D. R. & Miller, F. D. Trk signaling regulates neural precursor cell proliferation and differentiation during cortical development. *Development* **134**, 4369–4380 (2007). 18003743 10.1242/dev.008227

[32] Malatesta, P. et al. Neuronal or glial progeny: regional differences in radial glia fate. *Neuron* **37**, 751–764 (2003). 12628166 10.1016/s0896-6273(03)00116-8

[33] Brown, J. P. et al. Transient expression of doublecortin during adult neurogenesis. *J. Comp. Neurol.* **467**, 1–10 (2003). 14574675 10.1002/cne.10874

[34] Couillard-Despres, S. et al. Doublecortin expression levels in adult brain reflect neurogenesis. *Eur. J. Neurosci.* **21**, 1–14 (2005). 15654838 10.1111/j.1460-9568.2004.03813.x

[35] Jacobs, S. et al. Mice with targeted *Slc4a10* gene disruption have small brain ventricles and show reduced neuronal excitability. *Proc. Natl Acad. Sci. USA* **105**, 311–316 (2008). 18165320 10.1073/pnas.0705487105 PMC2224208

[36] Bastidas-Ponce, A. et al. Comprehensive single cell mRNA profiling reveals a detailed roadmap for pancreatic endocrinogenesis. *Development* **146**, dev173849 (2019). 31160421 10.1242/dev.173849

[37] Byrnes, L. E. et al. Lineage dynamics of murine pancreatic development at single-cell resolution. *Nat. Commun.* **9**, 3922 (2018). 30254276 10.1038/s41467-018-06176-3 PMC6156586

[38] Li, X. Y., Zhai, W. J. & Teng, C. B. Notch signaling in pancreatic development. *Int. J. Mol. Sci.* **17**, 48 (2015). 26729103 10.3390/ijms17010048 PMC4730293

[39] Hoffman, B. G., Zavaglia, B., Beach, M. & Helgason, C. D. Expression of Groucho/TLE proteins during pancreas development. *BMC Dev. Biol.* **8**, 81 (2008). 18778483 10.1186/1471-213X-8-81 PMC2551604

[40] Battich, N. et al. Sequencing metabolically labeled transcripts in single cells reveals mRNA turnover strategies. *Science* **367**, 1151–1156 (2020). 32139547 10.1126/science.aax3072

[41] Wolf, F. A., Angerer, P. & Theis, F. J. SCANPY: large-scale single-cell gene expression data analysis. *Genome Biol.* **19**, 15 (2018). 29409532 10.1186/s13059-017-1382-0 PMC5802054

[42] Gorin, G., Fang, M., Chari, T. & Pachter, L. RNA velocity unraveled. *PLoS Comput. Biol.* **18**, e1010492 (2022). 36094956 10.1371/journal.pcbi.1010492 PMC9499228

[43] Jung, M. & Lee, E. K. RNA-binding protein HuD as a versatile factor in neuronal and non-neuronal systems. *Biolog (Basel)* **10**, 361 (2021). 10.3390/biology10050361 PMC8145660 33922479

[44] Gao, M., Qiao, C. & Huang, Y. UniTVelo: temporally unified RNA velocity reinforces single-cell trajectory inference. *Nat. Commun.* **13**, 6586 (2022). 10.1038/s41467-022-34188-7 PMC9633790 36329018

[45] Gorin, G., Svensson, V. & Pachter, L. Protein velocity and acceleration from single-cell multiomics experiments. *Genome Biol.* **21**, 39 (2020). 32070398 10.1186/s13059-020-1945-3 PMC7029606

[46] Buenrostro, J. D. et al. Single-cell chromatin accessibility reveals principles of regulatory variation. *Nature* **523**, 486–490 (2015). 26083756 10.1038/nature14590 PMC4685948

[47] Hochreiter, S. & Schmidhuber, J. Long short-term memory. *Neural Comput.* **9**, 1735–1780 (1997). 9377276 10.1162/neco.1997.9.8.1735

[48] Lecun, Y., Bottou, L., Bengio, Y. & Haffner, P. Gradient-based learning applied to document recognition. *Proc. IEEE* **86**, 2278–2324 (1998).

[49] Paszke, A. et al. PyTorch: an imperative style, high-performance deep learning library. In *Advances in Neural Information Processing Systems* (Eds. Wallach, H. et al.) (NeurIPS, 2019).

[50] Virtanen, P. et al. SciPy 1.0: fundamental algorithms for scientific computing in Python. *Nat. Methods* **17**, 261–272 (2020). 32015543 10.1038/s41592-019-0686-2 PMC7056644

[51] Dormand, J. R. & Prince, P. J. A family of embedded Runge–Kutta formulae. *J. Comput. Appl. Math.* **6**, 19–26 (1980).

[52] Shampine, L. F. Some practical Runge–Kutta formulas. *Math. Comput.* **46**, 135–150 (1986).

[53] Hochgerner, H., Zeisel, A., Lonnerberg, P. & Linnarsson, S. Conserved properties of dentate gyrus neurogenesis across postnatal development revealed by single-cell RNA sequencing. *Nat. Neurosci.* **21**, 290–299 (2018). 29335606 10.1038/s41593-017-0056-2

