# Supplementary material for: A relay velocity model infers cell-dependent RNA velocity
Source: Nat Biotechnol. Author manuscript; Available in PMC 2024 Feb 16. (PMC10545816; doi:10.1038/s41587-023-01728-5)
Supplement: Supplementary Information [file NIHMS1899161-supplement-Supplementary_Information.pdf]

nature biotechnology

Article https://doi.org/10.1038/s41587-023-01728-5

A relay velocity model infers cell-dependent RNA velocity

In the format provided by the authors and unedited

Table of Contents

Supplementary Figures 2

Supplementary Fig. 1. The progress of minimizing the loss function. 2

Supplementary Fig. 2. RNA velocity estimation for branching genes in the hippocampal neurogenesis dataset. 3

Supplementary Fig. 3. α, β, and γ are indicators of cell identity. 4

Supplementary Notes 5

Supplementary Note 1: Develop a model-based neural network for RNA velocity inference 5

Supplementary Tables 8

Supplementary Table 1. The means of error rates of simulated transcriptional boost genes, multi-forward branching genes, and multi-backward branching genes using cellDancer, scVelo, velocyto, DeepVelo, and VeloVAE. 8

Supplementary Figures

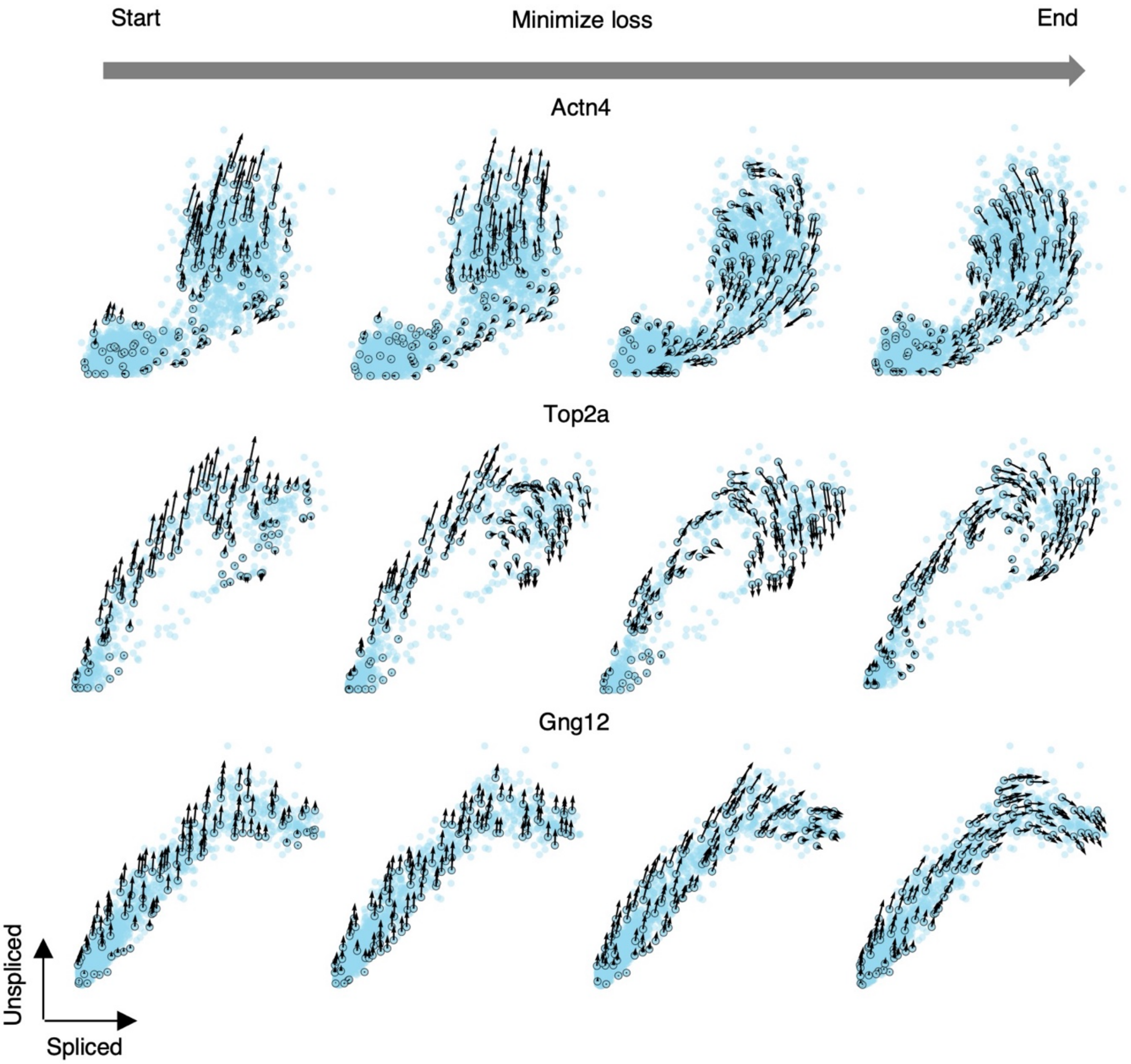

Supplementary Fig. 1. The progress of minimizing the loss function.

RNA velocities of three example genes (*Actn4*, *Top2a*, and *Gng12*) from the pancreatic endocrinogenesis cells are projected on their phase portraits along the training process of the DNNs.

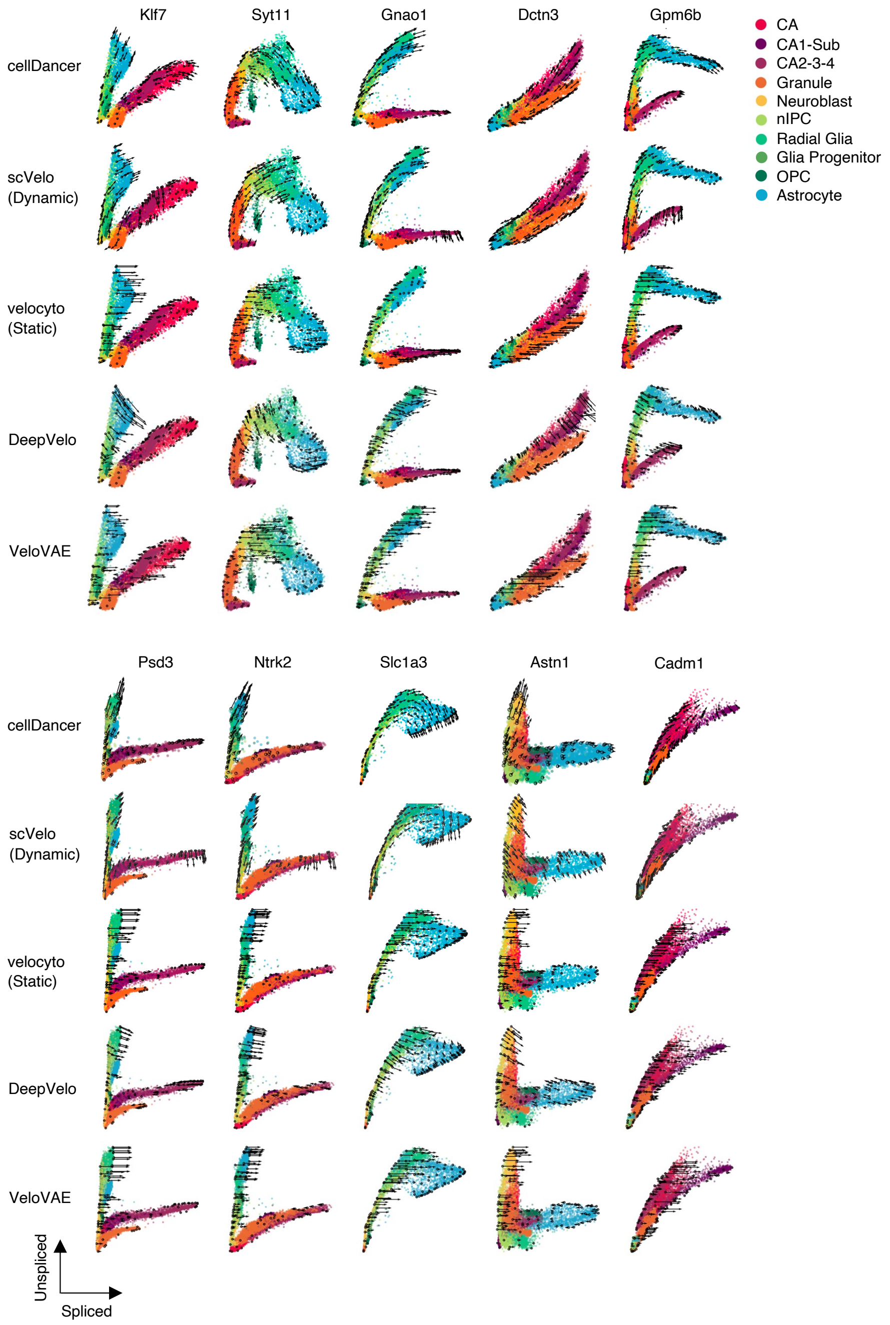

Supplementary Fig. 2. RNA velocity estimation for branching genes in the hippocampal neurogenesis dataset.

Comparison among the RNA velocities estimated by cellDancer, scVelo (dynamic model), velocyto (static model), DeepVelo, and VeloVAE in the hippocampal neurogenesis dataset. cellDancer outperforms the other four models for branching genes.

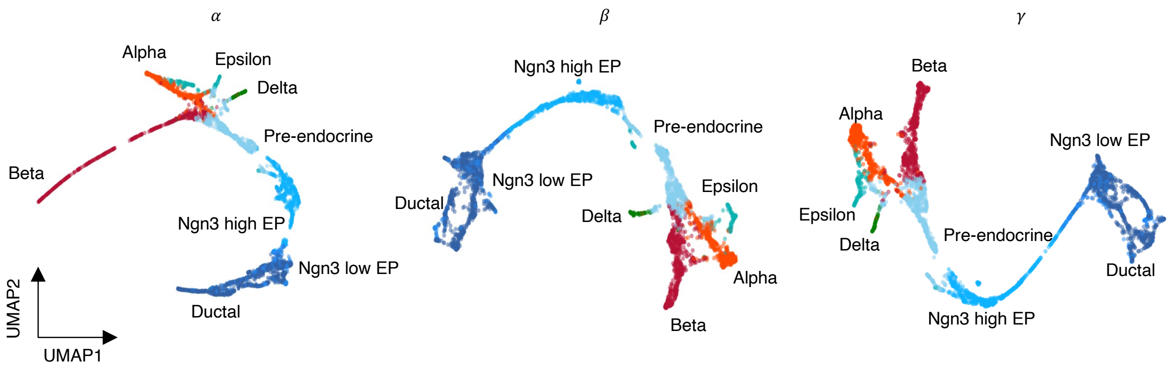

Supplementary Fig. 3. *α, β,* and *γ* are indicators of cell identity.

The UMAP embedding generated using the *α* (left), *β* (middle), and *γ* (right) in the pancreatic endocrinogenesis dataset shows that cell-dependent kinetic rates predicted by cellDancer can distinguish the cell sub-populations.

Supplementary Notes

Supplementary Note 1: Develop a model-based neural network for RNA velocity inference

To demonstrate the capability of the deep neural network (DNN) in RNA velocity inference, we rewrite the dynamic model of scVelo by adding a Heaviside step function (Note Figure 1A) to calculate transcription (α) rate as follows:

$$\frac{du}{dt} = \sigma(\beta u - \gamma s) * \alpha - \beta u$$
$$\frac{ds}{dt} = \beta u - \gamma s \qquad \text{Eqn. (1)}$$

where $\sigma(x) \equiv \begin{cases} 1, & x \geq 0 \\ 0, & x < 0 \end{cases}$. The sigmoid function is a widely used activation function in DNN. As the sigmoid function $s(x) = \frac{1}{1+e^{-1000x}}$ behaves similarly with the step function except for the former is differentiable, we can approximate $\sigma(x)$ with $s(x)$ in Eqn. (1) and solve Eqn. (1) by developing a simple prototype neural network using the sigmoid function.

First, we build a simplified neural network (Note Figure 1B) which has an input layer, a hidden layer, and an output layer. We separately train a network for each gene. We assume gene-specific constant values of α, β, and γ. Next, we develop a workflow to solve Eqn. (1) by optimizing the network as follows:

(1) For a given gene, the normalized (across all the cells) abundances of the unspliced and spliced mRNA (u_i and s_i) for one cell i are input to the network (Note Figure 1B). Weights $\{w\}$ and biases $\{b\}$ are applied to the input through a hidden layer to get ($w_\beta u_i + b_1, w_\gamma s_i + b_2$). Specifically in this simplified demonstration neural network, the weights are analogs of β and γ (($w_\beta \sim \beta$ and $w_\gamma \sim -\gamma$), and the biases are 0.

(2) Apply an activation function σ to the hidden layer outputs to obtain the relative reaction rates: $\tilde{\alpha}^i = \sigma(w_\beta u_i + w_\gamma s_i)$; $\tilde{\beta}$ and $\tilde{\gamma}$ are extracted from the weights $\tilde{\beta} = w_\beta$, $\tilde{\gamma} = -w_\gamma$.

(3) Predict the abundances at the next time step $u_{i,\text{next}} = (\tilde{\alpha}^i - \tilde{\beta}u_i)\Delta t + u_i$ and $s_{i,\text{next}} = (\tilde{\beta}u_i - \tilde{\gamma}s_i)\Delta t + s_i$ using the discretized equations from Eqn. (1). Then we compute the predicted displacement vector $\boldsymbol{v}_i^{\text{pred}} = (u_{i,\text{next}} - u_i, s_{i,\text{next}} - s_i)$ and estimate the loss function for cell i, $\mathcal{L}^i(u_i, s_i) = 1 - corr\left(\boldsymbol{v}_i^{\text{pred}}, \boldsymbol{v}_i^{\text{input}}\right)$, where the ground truth displacement $\boldsymbol{v}_i^{\text{input}}$ is obtained by a neighboring cell i' in the $\{u, s\}$ space which maximizes the correlation between the predicted vector and the input vector $\boldsymbol{v}_{ii'}^{\text{input}} = (u_{i'} - u_i, s_{i'} - s_i)$ as in the following equation: $i' = \underset{i'}{\text{argmax}}\, corr(\boldsymbol{v}_i^{\text{pred}}, \boldsymbol{v}_{ii'}^{\text{input}}) = \underset{i'}{\text{argmax}} \frac{\boldsymbol{v}_i^{\text{pred}} \cdot \boldsymbol{v}_{ii'}^{\text{input}}}{|\boldsymbol{v}_i^{\text{pred}}||\boldsymbol{v}_{ii'}^{\text{input}}|}$. The total loss for all the n cells is calculated as sum of the loss of individual cells, $\mathcal{L} = \sum_{i=1}^{n} \mathcal{L}^i\,(u_i, s_i)$.

(4) Adjust the $\{w\}$ and $\{b\}$ and repeat steps (2-3) to reduce the total loss $\mathcal{L}$ to yield the best estimates of the reaction rates.

We apply this network model to a simulation dataset for a gene following the dynamics in Eqn. (1) ($\alpha = 5.2, \beta = 2.0, \gamma = 1.0$; see details of the simulation in Methods). The initial guess was set to $\alpha = 1.0, \beta = 1.0, \gamma = 0.5$. We applied the adaptive gradient optimization algorithm Adam to minimize the total loss and the learning rate was 0.001. Results show that after each round (epoch) of training, prediction approaches closer to the ground truth (Note Figure 1C). In 1,500 epochs (within a few seconds), the prediction has converged and matches with the background truth, indicating the applicability of neural network to velocity estimation.

In practice, a more sophisticated deep neural network is constructed in cellDancer and trained to predict the reaction rates for a gene in all n cells $\{\tilde{\alpha}^i, \tilde{\beta}^i, \tilde{\gamma}^i\}_{i=1,2,\ldots,n}$ simultaneously. The network consists of an input layer with abundances of a gene from all the cells, two hidden layers with 100 nodes fully connected to all the input nodes, and an output layer with $3n$ using a sigmoid activation function (see Methods).

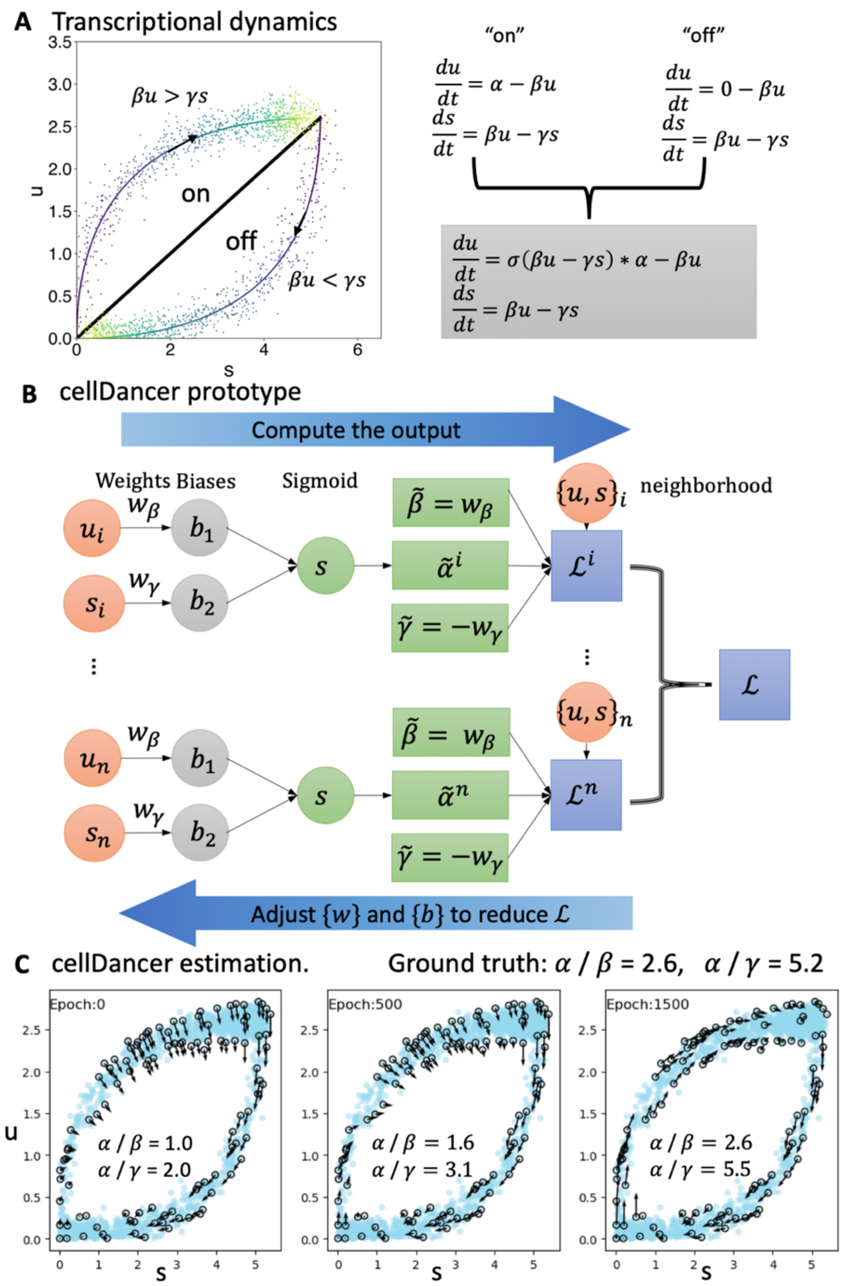

Note Figure 1. A model-based neural network for RNA velocity inference.

A. The transcriptional dynamics of a gene switching between the "on" and the "off" states are generalized with a Heaviside step function. **B**. A prototype neural network model demonstrates its suitability to learn the reaction rates. "u" and "s" indicate unspliced and spliced reads. **C**. cellDancer's network quickly converges to the ground truth in 1,500 epochs of training.

Supplementary Tables

	cellDancer	scVelo	velocyto	DeepVelo	VeloVAE
transcriptional boost	13.25%	46.88%	56.12%	51.62%	46.95%
multi-forward branching	2.63%	40.57%	67.67%	82.16%	58.82%
multi-backward branching	9.43%	31.13%	15.30%	44.99%	62.66%

Supplementary Table 1. The means of error rates of simulated transcriptional boost genes, multi-forward branching genes, and multi-backward branching genes using cellDancer, scVelo, velocyto, DeepVelo, and VeloVAE.
