# Supplementary material for: A relay velocity model infers cell-dependent RNA velocity
Source: Nat Biotechnol. 2023 Apr 3;42(1):99–108. doi: 10.1038/s41587-023-01728-5 (PMC10545816; doi:10.1038/s41587-023-01728-5)
Supplement: Supplementary file 1 [file 41587_2023_1728_MOESM2_ESM.pdf]

nature portfolio

Corresponding author(s): Guangyu Wang

Last updated by author(s): Feb 20, 2023

Reporting Summary

Nature Portfolio wishes to improve the reproducibility of the work that we publish. This form provides structure for consistency and transparency in reporting. For further information on Nature Portfolio policies, see our Editorial Policies and the Editorial Policy Checklist.

Statistics

For all statistical analyses, confirm that the following items are present in the figure legend, table legend, main text, or Methods section.

n/a	Confirmed	
☐	☒	The exact sample size (*n*) for each experimental group/condition, given as a discrete number and unit of measurement
☒	☐	A statement on whether measurements were taken from distinct samples or whether the same sample was measured repeatedly
☐	☒	The statistical test(s) used AND whether they are one- or two-sided<br>*Only common tests should be described solely by name; describe more complex techniques in the Methods section.*
☒	☐	A description of all covariates tested
☐	☒	A description of any assumptions or corrections, such as tests of normality and adjustment for multiple comparisons
☐	☒	A full description of the statistical parameters including central tendency (e.g. means) or other basic estimates (e.g. regression coefficient) AND variation (e.g. standard deviation) or associated estimates of uncertainty (e.g. confidence intervals)
☐	☒	For null hypothesis testing, the test statistic (e.g. *F*, *t*, *r*) with confidence intervals, effect sizes, degrees of freedom and *P* value noted<br>*Give P values as exact values whenever suitable.*
☒	☐	For Bayesian analysis, information on the choice of priors and Markov chain Monte Carlo settings
☒	☐	For hierarchical and complex designs, identification of the appropriate level for tests and full reporting of outcomes
☐	☒	Estimates of effect sizes (e.g. Cohen's *d*, Pearson's *r*), indicating how they were calculated

Our web collection on statistics for biologists contains articles on many of the points above.

Software and code

Policy information about availability of computer code

Data collection	No software was used for data collection.
Data analysis	The main functions of our study are open-source and could be obtained through https://github.com/GuangyuWangLab/cellDancer.<br>The comparison between our algorithm and other algorithms was performed by using cellDancer (v1.1.3), scVelo (v0.2.4), DeepVelo (v0.2.5rc1), VeloVAE (v0.1.0; under commit number 018ee60998c9b63f441fdfc4836dbd5baadf1459; https://github.com/welch-lab/VeloVAE).<br>The GO enrichment analysis was performed by DAVID (v2021.Dec).<br>The analysis of Rates-based UMAP and Expression-based UMAP of scEU-seq was based on SCANPY (v1.8.2).<br>The network and Adam optimizer of our software were implemented based on PyTorch Lightning (v1.5.2).<br>The Runge-Kutta method we used to solve differential equations in our simulation was performed by SciPy (v1.7.2).

For manuscripts utilizing custom algorithms or software that are central to the research but not yet described in published literature, software must be made available to editors and reviewers. We strongly encourage code deposition in a community repository (e.g. GitHub). See the Nature Portfolio guidelines for submitting code & software for further information.

Data

Policy information about availability of data

All manuscripts must include a data availability statement. This statement should provide the following information, where applicable:

- Accession codes, unique identifiers, or web links for publicly available datasets
- A description of any restrictions on data availability
- For clinical datasets or third party data, please ensure that the statement adheres to our policy

All the scRNA-seq raw data are publicly accessible. The pancreatic endocrinogenesis data can be extracted using scVelo's CLI: scvelo.datasets.pancreas() or accessed from the original work under accession number GSE132188 of Gene Expression Omnibus (GEO) repository. The hippocampal dentate gyrus neurogenesis data can be accessed from the link http://pklab.med.harvard.edu/velocyto/DentateGyrus/DentateGyrus.loom or the original paper under accession number GSE95753 of GEO. The erythroid lineage of mouse gastrulation data can be extracted using scVelo's CLI: scvelo.datasets.gastrulation() or from the original work under accession number E-MTAB-6967 of ArrayExpress. Human embryo glutamatergic neurogenesis can be accessed from https://github.com/pachterlab/GFCP_2022/blob/main/notebooks/data/hgForebrainGlut.loom or from the original work under accession code of Sequence Read Archive (SRA) SRP129388. Cell cycle progression in REP1-FUCCI cells can be extracted using dynamo's CLI: dyn.sample_data.scEU_seq_rpe1() or from the original work under accession number GSE128365 of GEO.

Human research participants

Policy information about studies involving human research participants and Sex and Gender in Research.

Reporting on sex and gender	No human research participants in this study.
Population characteristics	No human research participants in this study.
Recruitment	No human research participants in this study.
Ethics oversight	No human research participants in this study.

Note that full information on the approval of the study protocol must also be provided in the manuscript.

Field-specific reporting

Please select the one below that is the best fit for your research. If you are not sure, read the appropriate sections before making your selection.

☒ Life sciences ☐ Behavioural & social sciences ☐ Ecological, evolutionary & environmental sciences

For a reference copy of the document with all sections, see nature.com/documents/nr-reporting-summary-flat.pdf

Life sciences study design

All studies must disclose on these points even when the disclosure is negative.

Sample size	Sample sizes were determined in originally published publications. Mouse gastrulation erythropoiesis data: n=12,329 cells; mouse hippocampus development data: n=18,140 cells; mouse pancreas endocrine development data: n=3,696 cells; scEU-seq data: n=3,058 cells; human embryonic glutamatergic neurogenesis data: n=1,054 cells.
Data exclusions	No data were excluded.
Replication	No experiments in this study.
Randomization	No experiments in this study.
Blinding	No experiments in this study.

Reporting for specific materials, systems and methods

We require information from authors about some types of materials, experimental systems and methods used in many studies. Here, indicate whether each material, system or method listed is relevant to your study. If you are not sure if a list item applies to your research, read the appropriate section before selecting a response.

Materials & experimental systems

n/a	Involved in the study	
☒	☐	Antibodies
☒	☐	Eukaryotic cell lines
☒	☐	Palaeontology and archaeology
☒	☐	Animals and other organisms
☒	☐	Clinical data
☒	☐	Dual use research of concern

Methods

n/a	Involved in the study	
☒	☐	ChIP-seq
☒	☐	Flow cytometry
☒	☐	MRI-based neuroimaging
